# The economic burden of rheumatic heart disease in the Eastern Mediterranean Region

**DOI:** 10.1136/bmjgh-2024-018277

**Published:** 2025-10-07

**Authors:** Khalifa Elmusharaf, Sébastien Poix, Emil Basil Scaria, Mariyam Saherwala, Matilda Byström, Asmus Hammerich, Abdirahman Khalif Mohamud, Hicham El Berri, Eiman Hag

**Affiliations:** 1 Public Health, University of Birmingham Dubai, Dubai, UAE; 2 School of Medicine, University of Limerick, Limerick, Ireland; 3 World Health Organisation Regional Office for the Eastern Mediterranean, Cairo, Egypt

**Keywords:** Cardiovascular disease, Health economics

## Abstract

**ABSTRACT:**

**Introduction:**

Acute rheumatic fever (ARF) is an immune response triggered by group A Streptococcus (GAS) infections, predominantly affecting children aged 5–15 years. Untreated ARF can progress to rheumatic heart disease (RHD), causing complications such as valve stenosis, heart failure and stroke. Despite preventability using antibiotics, RHD persists as a major health concern in many Eastern Mediterranean Region (EMR) countries. Beyond its health implications, RHD poses a significant economic burden on societies, emphasising the need for comprehensive solutions.

**Methods:**

An economic analysis was conducted to estimate the burden of RHD in 22 countries of the EMR using data from secondary databases and existing research. The analysis modelled the future disease burden, using a scenario of inaction and considering population growth and changes in population structure. Economic losses associated with RHD were explored through five pathways, including the (a) direct healthcare costs of preventing and treating RHD and its complications, (b) cost of premature deaths, (c) cost of absenteeism in adult patients, (d) cost of absenteeism in caregivers and (e) future productivity losses due to unfulfilled potential in children.

**Results:**

The estimated economic burden of ARF, RHD and its related complications in the EMR was US$5.8 billion in 2020, amounting to 0.20% of the combined gross domestic product. Indirect costs accounted for 78% of the burden. 192 million prevalent cases and 1.5 million deaths were expected by 2050. The burden is projected to increase to US$166 billion between 2020 and 2050.

**Conclusion:**

The disease burden of RHD is rising in the EMR, widening health inequalities and straining resources. Careful planning and resource allocation based on economic evaluations are crucial to mitigate this issue. Regional governments must implement measures to address social determinants, enhance community awareness, improve surveillance and provide accessible healthcare services to effectively tackle this challenge.

**WHAT IS ALREADY KNOWN ON THIS TOPIC**

- Rheumatic heart disease (RHD) poses a significant health and economic burden to the world.
- RHD is a preventable condition, and cost-effective prevention programmes must be devised based on economic evaluations.
- The prevalence, mortality and economic burden of RHD varies within the countries of the Eastern Mediterranean Region (EMR) and needs to be studied in detail to develop strategies for prevention.

**WHAT THIS STUDY ADDS**

- The total economic burden of acute rheumatic fever (ARF), RHD and its common complications in EMR countries in 2020 is estimated to be US$5.8 billion, which is 0.20% of the total gross domestic product.
- Indirect costs, including costs due to premature deaths, absenteeism of adult patients and caregivers and loss of future productivity due to unfulfilled potential in children, account for 78% of the burden.
- Between 2020 and 2050, RHD is expected to cost the EMR countries US$166 billion.

**HOW THIS STUDY MIGHT AFFECT RESEARCH, PRACTICE OR POLICY**

- RHD significantly contributes to productivity loss and strains healthcare resources, placing a substantial burden on the economies of EMR countries.
- RHD burden is expected to increase in the next decades, and the development of comprehensive solutions aligning with the framework for action on RF and RHD developed by the WHO Regional Office for the Eastern Mediterranean can help reduce this burden.
- There is a need for country-level economic evaluations using local data to accurately assess the burden due to RHD and develop action plans, especially in lower-income countries.

## Introduction

 Acute rheumatic fever (ARF) is an abnormal immunologic response to group A Streptococcus (GAS) infections arising as a sequela of untreated pharyngitis. It manifests 2–3 weeks after the onset of infection and may have cardiac, neurologic, musculoskeletal or dermatological manifestations.^1 2^ While ARF is common in children aged 5–15 years, it can affect individuals of every age. ARF has a high recurrence rate, particularly in patients left untreated. Severe or recurrent bouts of ARF can result in the development of complications such as rheumatic heart disease (RHD), which is caused by molecular mimicry. As the streptococcal M-protein bears structural resemblance to cardiac myosin and valvular endothelium, the immune system mistakenly targets cardiac tissues, causing valvular damage and disruption of cardiac function.^3^ This may lead to various heart-related issues, such as arrhythmias, heart failure, stroke and infective endocarditis. There is no cure for RHD, and treatment is symptomatic. In case there is damage to the heart valves, surgical intervention is carried out to replace or repair the damaged valves.^4^ RHD in pregnancy can cause both maternal and fetal complications, with increased risk of preterm delivery, abortions and intrauterine death.^5^ RHD is a preventable condition, primarily through the prompt and effective treatment of streptococcal infections with antibiotics, such as penicillin. Additionally, regular follow-up and treatment by healthcare providers can help manage RHD and reduce its impact on affected individuals.^1^

The economic burden of RHD is a complex and pervasive challenge. At its core, RHD generates direct costs as individuals and their families grapple with medical expenses related to outpatient care, hospitalisation, medication and diagnostics. These ‘out-of-pocket’ expenses often drive individuals into medical poverty, perpetuating cycles of financial hardship. Socioeconomic factors such as poverty, undernutrition, poor housing and overcrowding contribute further to this vicious cycle.^6^ The Global Rheumatic Heart Disease Registry (REMEDY study) found that utilisation of secondary prophylaxis was suboptimal, and there were significant differences in the treatment of RHD and its complications based on the income level of countries.^7^ A study conducted in Uganda found that the economic burden of RHD had a catastrophic impact on the household income of those affected, with the direct and indirect costs amounting to US$78 per patient annually.^8^ The average direct cost per patient in South Africa in 2021 was found to be US$3900, significantly higher than many other infectious and non-communicable diseases.^9^ Recent data from Africa suggest that there is a high prevalence of subclinical RHD in Africa, and primary prevention of ARF is the most cost-effective strategy in these countries.^10^

Beyond direct costs, RHD imposes indirect burdens, primarily through the long-term reduction in social and economic participation. Parents may forego work to provide care, leading to income loss and, in some cases, unemployment. Moreover, the intangible costs of RHD, such as the anxiety and diminished quality of life experienced by those with the disease, compound the economic impact. A modelling study using data from 107 developing countries found that premature death was the primary cause of the economic burden of RHD.^11^ A study estimating the burden of RHD in the Middle East and North African regions between 1990 and 2019 found that while the overall burden of disease declined, drastic socioeconomic changes in the region continue to place a significant economic burden, especially in endemic areas.^12^ Children with RHD may struggle to complete their education on time. This lower school attainment translates into a future loss of productivity, and their potential contributions to the workforce are compromised.^13^ Thus, RHD’s economic consequences extend beyond the immediate healthcare costs and income loss to encompass the future potential productivity losses among children.

RHD’s societal impact extends to healthcare systems and national budgets, particularly in countries with subsidised healthcare. Addressing RHD in the Eastern Mediterranean Region (EMR) necessitates a forward-looking approach rooted in projection and planning. A consultative meeting between the World Heart Federation and the WHO Regional Office for the Eastern Mediterranean (WHO EMRO) to discuss an action plan for RHD control reiterated the need for research to quantify and assess the RHD burden. By projecting the economic burden associated with RHD, we can provide invaluable insights to inform policy makers, healthcare providers and other stakeholders. The aim of this study is to estimate the current and future health and economic burden of RHD in the EMR between 2020 and 2050. The results can inform policy decisions and prioritisation to allocate adequate resources to curb its detrimental effects. This analysis will also aid in cross-country comparisons of RHD burden in the EMR.

## Methods

### Study design

This study is an economic burden analysis using secondary data. Five pathways were used to explore the economic impact associated with RHD, such as the (a) direct healthcare costs incurred in preventing and treating RHD and its complications, (b) cost of premature deaths, (c) cost of absenteeism in adult patients, (d) cost of absenteeism in caregivers and (e) future productivity losses due to unfulfilled potential in children. Epidemiological estimates from the Institute of Health and Metrics and Evaluation (IHME) Global Burden of Disease (GBD) 2019^14^ were used to determine incidence, prevalence and deaths due to RHD in the EMR countries (online supplemental tables 1A–C). Incidence, prevalence and mortality rates at baseline were kept constant and combined with population projections from the United Nations World Population Prospects (UN WPP) database to estimate the future burden of RHD.^15^ These projections simulate a status quo scenario in which changes in epidemiological patterns are only driven by population growth and modifications of the population structure, and the epidemiological rates are assumed to remain constant. The analysis was also conducted using the upper and lower intervals of all IHME epidemiological estimates (incidence, prevalence and mortality) to obtain uncertainty intervals.

### Selected countries

The health and economic burden of RHD was calculated for each country of the EMR. However, to facilitate the interpretation of the findings, the results are reported by groups of countries based on the following World Bank income classification. *High-income countries are* Bahrain, Kuwait, Oman, Qatar, Saudi Arabia and the United Arab Emirates. *Middle-income countries* include Egypt, Islamic Republic of Iran, Iraq, Jordan, Lebanon, Libya, Morocco, Occupied Palestinian territory, Syrian Arab Republic and Tunisia. *Low-income countries include* Afghanistan, Djibouti, Pakistan, Somalia, Sudan and Yemen. The Syrian Arab Republic was added to the group of middle-income countries due to fluctuation of their income level and contraction in the classification.

### Data sources

The data required to conduct this study were obtained from publicly accessible global databases and peer-reviewed literature on the RHD. Regional studies and estimates were used when available to improve the accuracy of findings. Online supplemental materials 2 contains a list of the data sources (online supplemental table 2) and assumptions used in this study.

### Procedures

This study estimated the current and future health and economic burden of RHD in the EMR between 2020 and 2050. It explored the economic impact associated with RHD through five pathways. The costs were reported in US$, and currency conversions were made as per exchange rates in 2020. The pathways and the calculation methods used for each are described below:

#### Direct costs

Direct costs pertain to expenses incurred within the healthcare system for treating (1) ARF, performing (2) valve surgery and treating common complications in patients affected by RHD, including (3) heart failure, (4) atrial fibrillation, (5) infective endocarditis and (6) stroke. These costs encompass medical staff salaries, equipment, diagnostic procedures and treatments.^16^ They were calculated as follows:


$$DC=\sum P_{i}\times {CR}_{i}\times {AC}_{i}$$

Where,

P is the number of individuals requiring intervention i,

CR is the coverage rate for intervention i,

AC is the average treatment cost for intervention i.

The number of individuals requiring each intervention was calculated in two steps. First, the annual number of individuals affected by RHD was obtained by combining prevalence rates from the IHME^14^ and population projections from the UN WPP database.^15^ The analysis was also conducted using the upper and lower intervals of prevalence estimates to obtain uncertainty intervals.

The population in need related to each intervention was then calculated by applying a factor derived from the scenario developed from a cross-sectional study and time series analysis conducted in Brazil in 2018.^17^ This study, in turn, was based on data from the REMEDY study, which was a large study published in 2015 that enrolled participants from several of the countries in the EMR. The treatment coverage rates for valve surgery were obtained from the REMEDY study.^7^ The WHO Universal Health Coverage subindexes for non-communicable diseases and infectious diseases were used to approximate the treatment coverage rates for RHD complications.^18^ The average treatment cost of ARF, valve surgery and poststroke was estimated using a cost-ingredient method. This approach calculated the average treatment cost as the sum of the costs related to (1) drugs and supplies, (2) outpatient visits and (3) inpatient visits. The drug and supply costs were extracted from the OneHealth Tool (OHT).^19^ The costs of outpatient and inpatient visits were calculated by multiplying assumptions on the average number of visits extracted from the OHT and average visit costs from the WHO-CHOICE (CHOosing Interventions that are Cost-Effective) database.^20^ The reference costs were the cost per outpatient visit at primary hospitals and the cost per day at secondary-level hospitals. The costs of inpatient and outpatient visits were adjusted for inflation using historical trends from the International Monetary Fund^21^ to reflect their value at the baseline year. Due to a lack of available information on the treatment costs related to atrial fibrillation, infective endocarditis and heart failure, we estimated these costs as a proportion of poststroke treatment costs using ratios derived from a study conducted in Brazil.^17^ The number of individuals requiring each intervention, average treatment costs and coverage rates per country are reported in online supplemental tables 3–5, respectively.

#### Indirect costs

##### Cost of absenteeism in adult patients

Excess absenteeism was defined as the average additional days of work that employees missed due to RHD. The foregone productivity due to absenteeism was calculated from the annual number of employees affected by RHD, the average excess days of absence from work and the gross domestic product (GDP) per worker.^22^ The annual cost of absenteeism was calculated as follows:


$$ABS=\left(C\times LFPR\right)\times \left(\frac{GDP}{LF}\right)\times PCR$$

Where,

C is the number of prevalent cases of RHD in individuals aged 15–69,

LFPR is the labour force participation rate,

GDP is the gross domestic product,

LF is the labour force,

PCR is the absenteeism-related productivity reduction coefficient.

The annual number of individuals affected by RHD was obtained by combining prevalence rates from the IHME^14^ and population projections from the UN WPP.^15^ The labour force participation rate and the total labour force were obtained from the International Labour Organisation (ILO) database.^23^ The 2020 GDP was obtained from the World Bank database.^24^ The total unproductive days due to absenteeism were ascertained from treatment regimens obtained from the OHT, which estimate an average of 12 days of inpatient/outpatient visits to treat RHD per year per patient.^19^ It is assumed that, at the very least, a patient misses work on the days that they require an outpatient or inpatient visit. This assumption is correlated with the results of a previous study indicating that patients with prior or established cardiovascular disease missed 11–12 working days per year due to hospitalisation.^25^ To estimate the absenteeism-related productivity reduction coefficient, the annual working days missed were divided by the average number of working days in each country.

##### Cost of absenteeism in caregivers

The cost of absenteeism in caregivers was calculated using the same method as for adult patients, assuming that, at the very least, a caregiver misses 12 working days per year, during which children require an outpatient or inpatient visit. The reference population was children aged 0–14, assuming all children affected by RHD required one caregiver. The labour force participation rate was also used to account for the fact that not all caregivers participate in the labour force.

##### Cost of premature deaths

The economic cost of premature deaths refers to the monetary value of the years of life lost (YLLs) due to RHD. This study used the value of a statistical life-year approach, a valuation of mortality risk reduction commonly used in economic burden analyses, to estimate the economic losses arising from RHD-related mortality. The annual net present value of the cost of premature deaths was calculated as follows:


$$CPM=\sum D_{a}\times \left(\frac{LE_{a}\times VSLY}{{\left(1+r\right)}^{LE_{a}}}\right)$$

Where,

D is the number of deaths for a 5-year age group *a*,

LE is the life expectancy at age a,

VSLY is the value of the statistical life-year,

r is the discount rate.

The annual deaths from RHD were obtained by combining mortality rates from the IHME^14^ and population projections from the UN WPP.^15^ The analysis was also conducted using the upper and lower intervals of mortality estimates to obtain uncertainty intervals.

Life expectancy by 5-year age group was obtained from the WHO Global Health Observatory.^26^ The value of a statistical life-year (VSLY) was calculated by dividing the estimated value of a statistical life in each country^27^ by the number of expected life years left among the median age of the workforce, extracted from the ILO database.^23^

The economic losses generated by RHD-caused mortality were discounted at 3%. The time horizon for discounting was defined by two variables. First, the residual life expectancy at the age of death, the latter being determined as the 5-year age group midpoint. Second, the difference between the year of the death and 2020, which was considered the baseline year.

##### Future loss of productivity due to unfulfilled potential

RHD during childhood is associated with fewer schooling years, fewer working opportunities and reduced earnings in the labour market at adult age, which ultimately results in diminished national productivity. This study used two assumptions regarding (a) the impact of RHD on schooling and (b) the economic consequences of reduced schooling. According to a study from Kenya, it was assumed that 40% of children and teenagers affected by RHD are behind by at least one grade.^28^ Using data from the OHT, it was assumed that 1 year of school loss translated into a decrease in future earnings between 0.70% and 8.30%, depending on the country (online supplemental table 6). The following formula was used to calculate future loss of productivity due to unfulfilled potential in children and teenagers:


$$FLP=\sum I_{a}\times 0.40\times LFPR\times \left(\frac{WY_{a}\times GDPW}{{\left(1+r\right)}^{WY_{a}}}\right)$$

Where,

I is the number of incident cases for a 5-year age group *a*,

0.40 is the proportion of children and teenagers assumed to be behind by at least one grade,

LFPR is the labour force participation rate,

WY is the number of potential working years in a lifetime for a 5-year age group *a,*

r is the discount rate.

The annual number of incident cases of RHD in children and teenagers aged 5–19 was obtained by combining mortality rates from the IHME^14^ and population projections from the UN WPP.^15^ The analysis was also conducted using the upper and lower intervals of incidence estimates to obtain uncertainty intervals. Incident cases instead of prevalent cases were used to avoid overestimation due to double-counting when reporting the cumulative economic burden throughout the study period. Sources and calculation methods to estimate the labour force participation rate and GDP per worker were detailed in a previous section. The working years in a lifetime were estimated at 54, assuming that children will work from 15 to 69. The future loss of productivity was discounted at 3%. The time horizon for discounting was defined by two variables. First, the number of working years in a lifetime, which was estimated at 54, based on the assumption that children will work from 15 to 69. Second, the difference between the year of the death and 2020, which was considered the baseline year.

##### Discounting

We adjusted the discounting period for each year between 2020 and 2050 to reflect the time elapsed between the year they were calculated and the baseline year (2020). This adjustment ensured that all economic losses presented in this analysis are expressed as their net present value in 2020.

## Results

### Total population

The population estimates for each group were estimated using data from the UN WPP.^15^ Low-income countries had the highest combined population (374.8 million), followed by middle-income countries (337.4 million). High-income countries had the lowest combined population with just over 53.6 million people.

### Current and future health burden of RHD

#### Mortality

**Figure 1:**
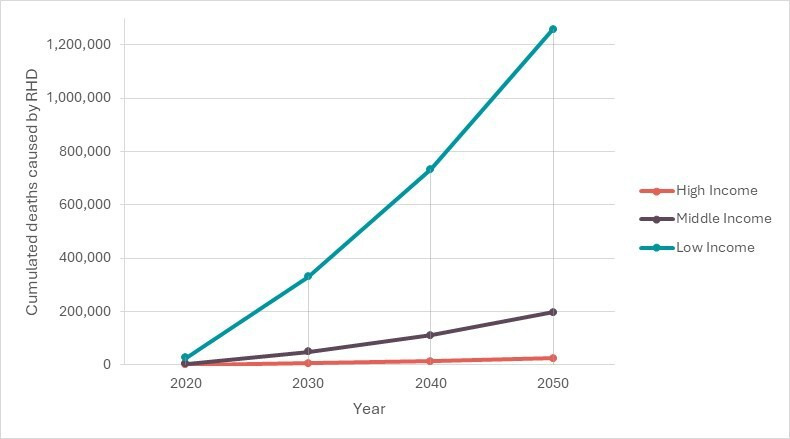
Cumulated number of RHD deaths (2020–2050) per country group. RHD, rheumatic heart disease.

Mortality due to RHD was estimated to cause 30 147 deaths in 2020, with the highest number of deaths occurring in low-income countries and the lowest in high-income countries (figure 1 and online supplemental table 7). The number of deaths is projected to increase in the next 30 years, with a cumulated total of 1.5 million deaths expected by 2050 in all the countries of the region combined.

#### Prevalence

**Figure 2:**
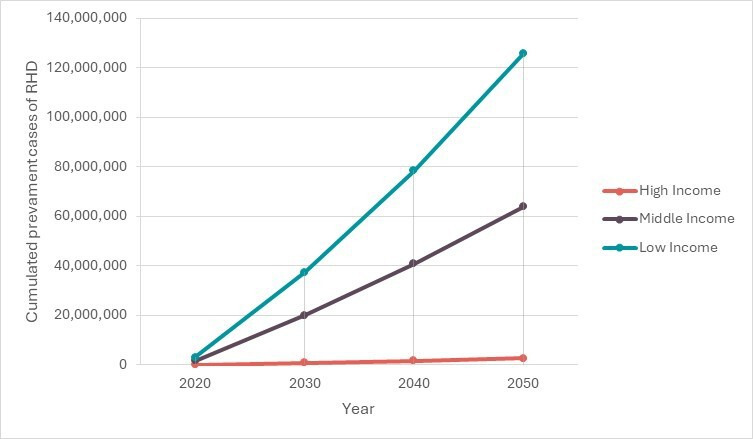
Cumulated number of prevalent cases (2020–2050) per country group. RHD, rheumatic heart disease.

The number of prevalent cases of RHD was 4.8 million in 2020 (figure 2 and online supplemental table 8). Low-income countries were the largest contributors (64% of cases). This trend was projected to continue until 2050, during which 192 million prevalent cases are expected in the region.

### Current and future economic burden of RHD

#### Total economic burden

The total economic burden of RHD in the EMR as of 2020 was US$5.8 billion. This is equivalent to 0.20% of the total GDP of the 22 countries in 2020 (table 1 and online supplemental table 9). Low-income countries had the highest economic burden at US$3 billion (0.80% of their combined GDPs), while high-income countries had a burden of US$1.4 billion (0.08% of their combined GDPs).

**Table 1:** Total economic burden as a percentage of the GDP in 2020 (in %) with uncertainty intervals

Year	High income (%)	Middle income (%)	Low income (%)	Total (% of 2020 GDP)
2020	0.08 (0.04–0.13)	0.15 (0.1–0.22)	0.8 (0.5–1.11)	0.2 (0.13–0.29)

GDP, gross domestic product.

**Figure 3:**
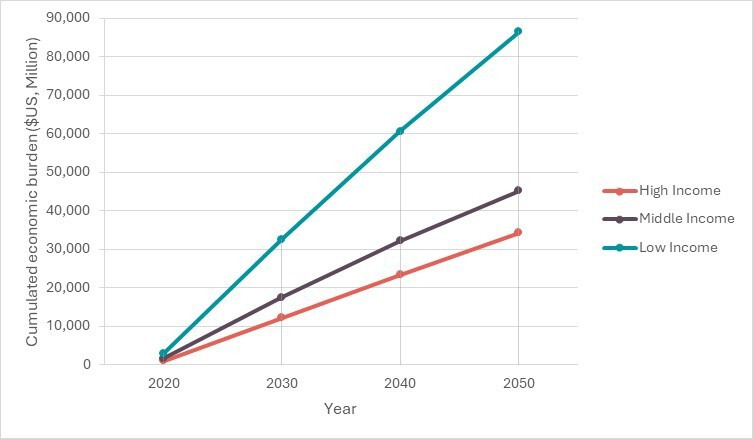
Cumulated economic burden in US$ (2020–2050) per country group.

In a status quo scenario, and considering future demographic changes, the total economic burden is projected to increase significantly to US$62.4 billion between 2020 and 2030, US$116.3 billion between 2020 and 2040 and US$166 billion between 2020 and 2050 (figure 3 and online supplemental table 10). The detailed economic burden by country and by pathway for the year 2020 and for the 2020–2050 period is reported in online supplemental tables 9 and 11, respectively.

### Economic burden by pathway

In 2020, the largest contributor to the economic burden was the cost of premature deaths, which is the monetary value of deaths that occur before reaching the expected life span. This was estimated to be 75% of the total economic burden (table 2).

**Table 2:** Contribution of the pathways to the total economic burden in 2020 (in millions, US$) with uncertainty intervals

Pathway	High income	Middle income	Low income	Total
Cost of premature deaths	889.6 (461.2–1515.3)	992.9 (689.9–1565.2)	2487.8 (1583.1–3590.4)	4370.3 (2734.2–6670.8)
Cost of absenteeism	53.8 (41.6–83.0)	51.1 (34.4–68.7)	22.2 (14.6–29.0)	127.1 (90.6–180.7)
Future productivity lost due to unfulfilled potential	11.5 (5.8–19.4)	19.5 (9.9–31.8)	20.0 (9.4–29.4)	51.0 (25.1–80.6)
Direct healthcare costs	152.6 (119.3–217.6)	596.4 (421.7–799.0)	500.8 (287.07–568.09)	1249.7 (828.1–1584.7)

Direct healthcare costs contributed to 21.6% of the economic burden. Overall, valve surgery was the intervention that produced the highest economic burden within this category, followed by treatment of heart failure (online supplemental table 12).

Costs due to absenteeism among patients and caregivers contributed 2.2% of the total economic burden, including 1.8% for adult patients and 0.4% for caregivers of children 0–14. The future loss of productivity due to unfulfilled potential contributed 0.9% of the total economic burden.

Based on out-of-pocket expenditure as a percentage of current health expenditure in each country, this study estimated that US$605 million was incurred by households in the EMR (online supplemental table 13).

### Sensitivity analysis

A sensitivity analysis was conducted adjusting the discount rates, which are used to calculate the present value of future costs (online supplemental table 14). By conducting the analysis using various discount rates, the range of possible outcomes can be better understood.

The discount rates were varied from 3% to 7%. At a discount rate of 3%, the total economic burden from 2020 to 2050 was estimated to be US$166 billion. When the discount rate was increased to 5%, the economic burden decreased to US$106.3 billion. When the discount rate was further increased to 7%, the total economic burden also decreased further to US$73 billion. The economic burden in 2020, as well as the economic burden as a percentage of GDP for each country group, is also provided in online supplemental table 14.

## Discussion

The findings indicate that RHD and its related complications cost an estimated US$5.8 billion to the countries of the EMR in 2020, representing 0.2% of the combined GDP. The projected total economic burden between 2020 and 2050 is US$166 billion, which denotes a significant burden to the regional economy. Low-income countries had the highest mortality, prevalence and total economic burden, while high-income countries had the least deaths, prevalent cases and economic burden due to RHD. The REMEDY study found major differences in the treatment of RHD based on the income level of the country. These disparities can be attributed to variations in populations, risk factors, discrepancies in treatment availability, differential levels of infection exposure and socioeconomic differences. Although the prevalence of patients with RHD and left ventricular (LV) dysfunction was higher in low- and lower-middle-income countries, the use of percutaneous and surgical interventions was extremely low compared with upper-middle-income countries. The relative lack of healthcare facilities and access could explain the variations between these countries in the suboptimal use of secondary prophylaxis and invasive interventions.^7^ This inequality in RHD disease and economic burden also aligns with previous studies that have found that RHD burden declines in countries with high sociodemographic index,^29^ and the greatest share of RHD disease burden is borne by low-income countries.^30^

The direct healthcare costs accounted for 21.6% of the total economic burden in 2020. Surgical intervention was the highest contributor to this (64%), which agrees with research from South Africa that attributed 65% of the direct costs to surgery.^9^ Valve repair, valve replacement and valvuloplasty are often required due to damage to the heart valves. Treatment costs for heart failure, ARF and stroke were the next highest contributors. It is evident that prevention and early detection can save significant costs due to RHD. Out-of-pocket expenditure due to RHD was found to constitute 11.1% of the total direct costs in the EMR (online supplemental table 15). It was highest in low-income countries at US$304 million (online supplemental table 16). Limited access to healthcare and the health insurance systems in poorer nations results in a vicious cycle of repeated bouts of RF and hospital admissions, which can further increase the economic burden placed on impoverished households.

Indirect costs contributed the largest share (78.4%) of the total economic burden. Similar studies across the world suggest that indirect costs are significantly higher when compared with direct costs.^31 32^ This could be because RHD commonly affects younger individuals, who suffer significant loss of productivity and decreased future earning potential due to the disease. Indirect costs contributed between 83% and 87% to the total economic burden in high- and low-income countries and 64% in middle-income countries. This could be because the direct healthcare costs were significantly higher in middle-income countries.

Premature deaths due to RHD were the main cost driver and contributed over 75% to the overall economic burden. This is expected, as costs due to premature deaths extend over several years, while direct healthcare costs and other indirect costs have more short-term impacts. Similar results were obtained by a study conducted in Fiji that estimated that premature deaths contributed 71.4% of the total costs.^32^ Between 2020 and 2050, a total of 1.5 million deaths due to RHD are projected in all the countries of the EMR combined.

In this study, we used the VSLY approach to estimate the economic burden of premature deaths caused by RHD in the EMR. The VSLY reflects the societal value of preventing premature deaths based on individuals’ willingness to pay to reduce mortality risks. By assigning a monetary value to all YLL, rather than only those occurring in the economically active population, this approach captures the intrinsic value of life, encompassing aspects such as emotional well-being, quality of life and societal welfare. Consequently, it provides a broader perspective of the impact of premature mortality that extends beyond mere productivity losses. It is a particularly relevant indicator in contexts where informal economies are prominent and where many women may not be part of the formal labour force, as is the case in several countries in the EMR. However, it is important to note that this method does not produce tangible costs, which limits direct comparisons with economic productivity-based indicators.

Previous research suggests that school absenteeism and failure are aggravated by ARF and RHD.^33^ Lower school attainment and poor performance often compromise the ability of patients with RHD to earn at their full potential and contribute to the economy.^13^ However, to our knowledge, the future productivity lost due to unfulfilled potential has not been quantified so far. This study calculated the future productivity loss due to unfulfilled potential and found that it accounts for 0.9% of the total economic burden. The challenges faced by caregivers of paediatric RHD patients have been explored by previous studies but were not quantified as an indirect cost.^34^ Parents and caregivers report poor quality of life and detrimental financial effects due to their loss of productivity.^35^ This study quantified the cost of absenteeism in caregivers and estimated that it contributed about 0.4% of the total economic burden.

A study of the cardiovascular disease burden in the EMR between 1990 and 2015 suggested that the age-standardised DALY was significantly higher compared with the global average, and RHD was one of the major contributors to this burden.^36^ As RHD is a chronic disease that cannot be cured, preventing the development of RHD from ARF may be the most cost-effective step, and current prevention programmes have shown marked success in reducing the incidence of RHD.^10^

### Limitations

This study has some limitations.

- Epidemiological data were derived from the IHME GBD 2019, introducing dependencies on the accuracy and completeness of estimates, which, in turn, rely on various data sources, methodologies and assumptions. However, the use of IHME estimates facilitates the study’s applicability across the 22 countries of the EMR, overcoming challenges related to the availability of national data sources. Moreover, updated epidemiological data were released by the IHME GBD for 2020 and 2021 after this analysis was completed, which could have a potential impact on findings.
- Due to challenges in measuring and projecting future epidemiological trends into the future, the projections only account for demographic shifts. Previous trends indicate that incidence, prevalence and mortality rates would increase in the future due to increases in risk factors. However, the effects of better preventive care, advancements in diagnosis and treatment could reverse these trends.
- Certain variables used to calculate the direct healthcare costs, such as the number of individuals requiring treatment for RHD comorbidities, the related treatment costs and coverage rates, are subject to a level of uncertainty. The estimation of these parameters relies on various sources, including previous studies and international databases. Differences in healthcare systems, epidemiological patterns and population characteristics across the EMR may introduce variability in the accuracy of these estimations. We also conducted the analysis using the upper and lower values of the epidemiological estimates from IHME to generate uncertainty intervals. These help account for uncertainty and provide a possible range for the final results.
- The model does not account for future changes in productivity indicators due to the considerable uncertainty surrounding these variables. Introducing assumptions was not considered feasible with such a large level of uncertainty.
- Another notable assumption in this study pertains to the impact of RHD on schooling, which was estimated based on a study conducted in Kenya. This study used a limited sample size and was conducted over a short period. Also, the educational landscape, socioeconomic conditions and healthcare infrastructure in Kenya may not be fully representative of high-income countries within the EMR. The generalisability of this assumption to countries with diverse cultural, economic and educational systems may introduce uncertainties in the projections of future productivity losses due to unfulfilled potential in children affected by RHD.
- When data were not available for a country, proxy indicators were chosen from neighbouring, socioeconomically similar countries. However, it must be noted that even seemingly similar countries can have significant differences.

### Recommendations

In 2018, resolution WHA71.14 was passed in the World Health Assembly to address the global burden of RF and RHD. It identified RHD as a preventable condition that could be averted with focused efforts from Member States and observed that RHD was caused by socioeconomic factors such as poor housing, poverty, overcrowding and reduced access to health services. It called on members to accelerate research to quantify the burden of disease, implement robust surveillance and data collection systems, develop prevention programmes and improve healthcare access. The need to design cost-effective diagnostic and treatment modalities was emphasised, especially in endemic areas.^6^ In line with this resolution, the WHO EMRO has developed a regional framework to tackle the burden of RHD in the region.^37^ It cites strong evidence to guide ARF/RHD control and detailed opportunities to intervene at every level of prevention. Some of its key recommendations include surveillance, development of diagnostic and treatment guidelines, provision of affordable and accessible healthcare, including valve surgeries, and focus on alleviating poor housing conditions. Increased community awareness and preparedness among healthcare workers also provide an effective pathway to reducing the RHD burden.

Prevention of RHD with prompt diagnosis and antibiotic treatment of ARF has shown effectiveness in reducing the incidence of this condition.^38^ These strategies can be implemented at primary, secondary and tertiary levels. At the primary prevention level, improvement in hygiene and sanitation can reduce the transmission of GAS infections. In remote Queensland, free mobile laundries have been introduced to improve hygiene among Aboriginal and Torres Strait Islander communities. This initiative has significantly reduced the incidence of RHD by addressing one of its root causes—poor hygiene due to limited access to laundry facilities.^39^ Provision of adequate housing can also prevent overcrowding and reduce disease incidence. Sudan implemented the SUR I CAAN framework, focusing on Surveillance, Integration, Collaboration, Awareness, Advocacy and Training. This comprehensive approach led to the integration of RHD control into national health programmes and improved early detection through electronic registries and echocardiographic screenings.^40^

At the secondary prevention level, regular screening programmes in high-risk populations can be an effective tool to reduce RHD burden. Antibiotics, such as penicillin, are used to prevent ARF, especially in cases of suspected GAS pharyngitis, a precursor to RHD.^41^ A systematic review revealed that antibiotic treatment of GAS throat infections can reduce the attack rate of ARF by 70%–80%, and the cost of preventing one case of ARF with a single intramuscular injection was US$46 in South Africa.^9^ A cost-effectiveness analysis found that combining echocardiography screening and long-term antibiotic prophylaxis was the most cost-effective method to reduce the overall RHD incidence.^42^ These preventive measures avoid permanent damage to cardiac tissue and valves, which are expensive to repair and contribute the highest percentage of direct healthcare costs. A Cuban study evaluating prevention programmes found that a combined primary and secondary prevention programme offered significant cost-saving benefits compared with a ‘do nothing’ approach. Over 10 years, the programme averted 1844 DALYs per 100 000 school-aged children and saved US$7 848 590 for a total programme cost of just US$202 890.^43^ Establishing surveillance systems such as electronic registries to monitor ARF and RHD can improve the timely provision of interventions. Nepal has conducted a successful RHD control programme, integrating RHD prevention and control into existing healthcare services. The programme includes community awareness campaigns, training for healthcare workers and regular monitoring, leading to improved management and prevention of RHD.^44^ To alleviate the economic impact of RHD, the following policies could be implemented:

- Establishment of robust surveillance systems to monitor RHD prevalence and outcomes, which can inform policy decisions and resource allocation.
- Incorporation of RHD prevention and management into primary healthcare to ensure early detection and treatment, reducing the need for costly tertiary care.
- Implementation of policies that reduce out-of-pocket expenditures for RHD patients to prevent catastrophic health spending and improve access to necessary services, such as insurance and financial assistance schemes.
- Raising awareness about the prevention and early signs of RHD through community-based campaigns.

Addressing the economic burden of RHD requires a multifaceted approach, including investment in prevention, strengthening healthcare systems and implementing supportive policies. By focusing on these strategies, governments and health organisations can improve health outcomes and reduce the financial impact of RHD on individuals and societies.

The comparison between different groups of countries in the EMR poses a challenge as they differ substantially in demographics, socioeconomic status and health systems, even within groups. Many of the countries in the region are impacted by emergencies such as conflict, political and/or financial instability or natural disasters, all of which have detrimental effects on the social, economic and health systems of the respective countries.^45^ Poverty is a fundamental driver of RHD, as it often leads to overcrowded living conditions, poor sanitation and limited access to healthcare. Our findings suggest that countries with lower income levels tend to have a higher burden from RHD.^46^ Overcrowding and inadequate housing conditions increase the transmission risk of GAS infections, with larger household sizes having been linked to higher RHD prevalence.^47^ Nutritional factors also play a role. Low-income countries with higher prevalence of anaemia and nutritional deficiencies had a higher prevalence and mortality due to RHD. In the EMR, disparities in healthcare infrastructure also contribute to the variability in RHD burden. For example, countries with a higher number of physicians per 1000 population tend to have lower RHD mortality rates. High-income countries, with a higher physician to population ratio, have lower burden from RHD.^48^ Furthermore, higher out-of-pocket healthcare expenditures are associated with increased RHD mortality, as financial hardship can deter individuals from seeking timely medical attention.^46^ Low-income countries, with the highest out-of-pocket healthcare expenditure, suffered the highest burden.

### Future research

There are gaps in public health research from the EMR, especially with regards to non-communicable diseases.^49^ Comprehensive longitudinal studies are essential to elucidate the natural history of RHD, from initial GAS infection to the development of ARF and subsequent RHD. More research to investigate the effectiveness of prevention strategies, such as prophylactic antibiotic treatment of GAS pharyngitis, is also essential. These studies can provide insights into disease progression, identify critical intervention points and inform the development of targeted prevention and treatment strategies.

Research to understand the barriers in programme delivery, resource allocation and community engagement can also help in developing more effective RHD control programmes, especially in the EMR. These studies can aid prioritisation of strategies and guide best practices and policy formulations.

There is a dearth of high-quality data for RHD indicators for many countries in the region, and further research is needed to plug this gap. This also raises the need for conducting such economic analyses at the country level to improve the quality of results. The widespread variations in economic and disease burden of RHD in the region also require careful resource allocation in endemic areas and countries with resource constraints.

## Conclusion

This study highlights the rising health and economic burden of RHD in the EMR, with a disproportionately severe impact on lower-income countries. It underscores the importance of careful resource allocation, guided by economic evaluations, to address the varying burdens within the region. Aligning with WHO recommendations, emphasis should be placed on addressing the social determinants of RHD, ensuring robust surveillance and accessible healthcare and increasing community awareness and preparedness. Efforts led by WHO, particularly through implementing a regional framework for action, showcase the commitment to evidence-based interventions and pave the way for sustainable initiatives to combat RHD and improve the overall health landscape in the EMR. The recommended next step would be to conduct a burden of disease survey in the region to better understand the needs and implications for the population.

## Supplementary material

10.1136/bmjgh-2024-018277 online supplemental file 1

## Data Availability

All data relevant to the study are included in the article or uploaded as supplementary information.
