# Supplementary material for: The economic burden of rheumatic heart disease in the Eastern Mediterranean Region
Source: BMJ Glob Health. 2025 Oct 7;10(10):e018277. doi: 10.1136/bmjgh-2024-018277 (PMC12506036; doi:10.1136/bmjgh-2024-018277)
Supplement: online supplemental file 1 [file bmjgh-10-10-s001.docx]

# SUPPLEMENTARY MATERIALS

**Supplementary Table 1A. Mortality rate estimates with uncertainty intervals from IHME GBD 2019**

| Country | Group | Age | year | Mortality rate (Per 100,000) | Upper value | Lower value |
| --- | --- | --- | --- | --- | --- | --- |
| Afghanistan | Low-income | <5 years | 2019 | 0.19 | 0.46 | 0.07 |
|  |  | 5-9 years | 2019 | 0.28 | 0.57 | 0.14 |
|  |  | 10-14 years | 2019 | 0.48 | 0.84 | 0.24 |
|  |  | 15-19 years | 2019 | 0.74 | 1.45 | 0.36 |
|  |  | 20-24 years | 2019 | 1.43 | 2.51 | 0.76 |
|  |  | 25-29 years | 2019 | 1.95 | 3.36 | 0.96 |
|  |  | 30-34 years | 2019 | 2.66 | 4.56 | 1.38 |
|  |  | 35-39 years | 2019 | 3.71 | 6.35 | 1.95 |
|  |  | 40-44 years | 2019 | 6.67 | 11.23 | 3.54 |
|  |  | 45-49 years | 2019 | 8.10 | 13.67 | 4.32 |
|  |  | 50-54 years | 2019 | 9.74 | 15.75 | 5.19 |
|  |  | 55-59 years | 2019 | 13.64 | 23.15 | 7.01 |
|  |  | 60-64 years | 2019 | 14.73 | 25.12 | 7.02 |
|  |  | 65-69 years | 2019 | 20.95 | 38.65 | 9.98 |
|  |  | 70-74 years | 2019 | 24.49 | 47.44 | 11.56 |
|  |  | 75-79 years | 2019 | 35.19 | 74.03 | 15.88 |
|  |  | 80-84 years | 2019 | 41.70 | 85.24 | 19.40 |
|  |  | 85+ years | 2019 | 62.51 | 132.44 | 28.73 |
| Bahrain | High-income | <5 years | 2019 | 0.02 | 0.04 | 0.01 |
|  |  | 5-9 years | 2019 | 0.06 | 0.09 | 0.03 |
|  |  | 10-14 years | 2019 | 0.08 | 0.12 | 0.05 |
|  |  | 15-19 years | 2019 | 0.17 | 0.25 | 0.11 |
|  |  | 20-24 years | 2019 | 0.22 | 0.36 | 0.13 |
|  |  | 25-29 years | 2019 | 0.16 | 0.25 | 0.09 |
|  |  | 30-34 years | 2019 | 0.17 | 0.25 | 0.10 |
|  |  | 35-39 years | 2019 | 0.19 | 0.28 | 0.11 |
|  |  | 40-44 years | 2019 | 0.36 | 0.55 | 0.23 |
|  |  | 45-49 years | 2019 | 0.56 | 0.87 | 0.32 |
|  |  | 50-54 years | 2019 | 0.64 | 0.99 | 0.40 |
|  |  | 55-59 years | 2019 | 1.09 | 1.59 | 0.70 |
|  |  | 60-64 years | 2019 | 1.72 | 2.62 | 1.14 |
|  |  | 65-69 years | 2019 | 3.24 | 4.61 | 2.25 |
|  |  | 70-74 years | 2019 | 5.07 | 7.13 | 3.45 |
|  |  | 75-79 years | 2019 | 11.05 | 16.07 | 7.65 |
|  |  | 80-84 years | 2019 | 16.33 | 23.42 | 11.39 |
|  |  | 85+ years | 2019 | 27.26 | 37.17 | 17.98 |
| Djibouti | Low-income | <5 years | 2019 | 0.22 | 0.54 | 0.08 |
|  |  | 5-9 years | 2019 | 0.26 | 0.55 | 0.11 |
|  |  | 10-14 years | 2019 | 0.49 | 0.95 | 0.22 |
|  |  | 15-19 years | 2019 | 0.56 | 1.07 | 0.25 |
|  |  | 20-24 years | 2019 | 0.39 | 0.75 | 0.16 |
|  |  | 25-29 years | 2019 | 0.35 | 0.66 | 0.15 |
|  |  | 30-34 years | 2019 | 0.38 | 0.74 | 0.17 |
|  |  | 35-39 years | 2019 | 0.45 | 0.87 | 0.20 |
|  |  | 40-44 years | 2019 | 0.62 | 1.21 | 0.27 |
|  |  | 45-49 years | 2019 | 0.67 | 1.24 | 0.33 |
|  |  | 50-54 years | 2019 | 1.27 | 2.18 | 0.64 |
|  |  | 55-59 years | 2019 | 2.29 | 3.77 | 1.17 |
|  |  | 60-64 years | 2019 | 3.88 | 6.35 | 2.02 |
|  |  | 65-69 years | 2019 | 7.33 | 11.82 | 4.20 |
|  |  | 70-74 years | 2019 | 10.28 | 16.18 | 6.17 |
|  |  | 75-79 years | 2019 | 13.60 | 21.35 | 8.13 |
|  |  | 80-84 years | 2019 | 18.47 | 28.52 | 10.84 |
|  |  | 85+ years | 2019 | 30.35 | 45.44 | 18.41 |
| Egypt | Middle-income | <5 years | 2019 | 0.36 | 0.86 | 0.14 |
|  |  | 5-9 years | 2019 | 0.36 | 0.72 | 0.19 |
|  |  | 10-14 years | 2019 | 0.53 | 0.91 | 0.28 |
|  |  | 15-19 years | 2019 | 0.50 | 0.88 | 0.26 |
|  |  | 20-24 years | 2019 | 0.50 | 0.93 | 0.23 |
|  |  | 25-29 years | 2019 | 0.59 | 1.06 | 0.29 |
|  |  | 30-34 years | 2019 | 0.61 | 1.08 | 0.31 |
|  |  | 35-39 years | 2019 | 0.69 | 1.18 | 0.36 |
|  |  | 40-44 years | 2019 | 1.06 | 1.81 | 0.56 |
|  |  | 45-49 years | 2019 | 1.32 | 2.22 | 0.67 |
|  |  | 50-54 years | 2019 | 1.98 | 3.52 | 0.97 |
|  |  | 55-59 years | 2019 | 3.24 | 5.65 | 1.55 |
|  |  | 60-64 years | 2019 | 4.23 | 7.24 | 2.02 |
|  |  | 65-69 years | 2019 | 7.08 | 11.82 | 3.45 |
|  |  | 70-74 years | 2019 | 8.78 | 13.55 | 4.85 |
|  |  | 75-79 years | 2019 | 13.57 | 20.33 | 7.88 |
|  |  | 80-84 years | 2019 | 17.39 | 25.47 | 10.36 |
|  |  | 85+ years | 2019 | 25.03 | 35.35 | 15.83 |
| Iran, Islamic Republic of | Middle-income | <5 years | 2019 | 0.09 | 0.14 | 0.06 |
|  |  | 5-9 years | 2019 | 0.15 | 0.19 | 0.12 |
|  |  | 10-14 years | 2019 | 0.20 | 0.24 | 0.17 |
|  |  | 15-19 years | 2019 | 0.24 | 0.28 | 0.20 |
|  |  | 20-24 years | 2019 | 0.26 | 0.32 | 0.21 |
|  |  | 25-29 years | 2019 | 0.29 | 0.35 | 0.25 |
|  |  | 30-34 years | 2019 | 0.33 | 0.40 | 0.28 |
|  |  | 35-39 years | 2019 | 0.42 | 0.51 | 0.35 |
|  |  | 40-44 years | 2019 | 0.68 | 0.82 | 0.59 |
|  |  | 45-49 years | 2019 | 0.88 | 1.03 | 0.75 |
|  |  | 50-54 years | 2019 | 1.22 | 1.44 | 1.03 |
|  |  | 55-59 years | 2019 | 2.30 | 2.71 | 1.92 |
|  |  | 60-64 years | 2019 | 3.12 | 3.68 | 2.63 |
|  |  | 65-69 years | 2019 | 5.03 | 5.91 | 4.30 |
|  |  | 70-74 years | 2019 | 6.93 | 7.98 | 5.86 |
|  |  | 75-79 years | 2019 | 12.01 | 13.91 | 9.65 |
|  |  | 80-84 years | 2019 | 16.26 | 18.78 | 12.87 |
|  |  | 85+ years | 2019 | 28.10 | 32.74 | 21.58 |
| Iraq | Middle-income | <5 years | 2019 | 0.08 | 0.14 | 0.03 |
|  |  | 5-9 years | 2019 | 0.06 | 0.09 | 0.04 |
|  |  | 10-14 years | 2019 | 0.09 | 0.15 | 0.05 |
|  |  | 15-19 years | 2019 | 0.13 | 0.20 | 0.08 |
|  |  | 20-24 years | 2019 | 0.17 | 0.28 | 0.10 |
|  |  | 25-29 years | 2019 | 0.27 | 0.44 | 0.15 |
|  |  | 30-34 years | 2019 | 0.40 | 0.65 | 0.24 |
|  |  | 35-39 years | 2019 | 0.57 | 0.91 | 0.34 |
|  |  | 40-44 years | 2019 | 1.07 | 1.68 | 0.64 |
|  |  | 45-49 years | 2019 | 1.61 | 2.43 | 1.03 |
|  |  | 50-54 years | 2019 | 2.12 | 3.16 | 1.37 |
|  |  | 55-59 years | 2019 | 3.01 | 4.33 | 1.96 |
|  |  | 60-64 years | 2019 | 3.35 | 4.65 | 2.16 |
|  |  | 65-69 years | 2019 | 5.16 | 7.01 | 3.52 |
|  |  | 70-74 years | 2019 | 6.29 | 8.39 | 4.33 |
|  |  | 75-79 years | 2019 | 9.87 | 13.37 | 7.12 |
|  |  | 80-84 years | 2019 | 15.25 | 19.97 | 11.18 |
|  |  | 85+ years | 2019 | 26.38 | 33.51 | 19.34 |
| Jordan | Middle-income | <5 years | 2019 | 0.01 | 0.03 | 0.01 |
|  |  | 5-9 years | 2019 | 0.03 | 0.04 | 0.02 |
|  |  | 10-14 years | 2019 | 0.05 | 0.08 | 0.03 |
|  |  | 15-19 years | 2019 | 0.06 | 0.09 | 0.04 |
|  |  | 20-24 years | 2019 | 0.07 | 0.11 | 0.04 |
|  |  | 25-29 years | 2019 | 0.09 | 0.14 | 0.06 |
|  |  | 30-34 years | 2019 | 0.11 | 0.17 | 0.07 |
|  |  | 35-39 years | 2019 | 0.17 | 0.25 | 0.11 |
|  |  | 40-44 years | 2019 | 0.27 | 0.38 | 0.18 |
|  |  | 45-49 years | 2019 | 0.30 | 0.43 | 0.20 |
|  |  | 50-54 years | 2019 | 0.50 | 0.72 | 0.34 |
|  |  | 55-59 years | 2019 | 0.84 | 1.18 | 0.57 |
|  |  | 60-64 years | 2019 | 0.85 | 1.20 | 0.57 |
|  |  | 65-69 years | 2019 | 1.91 | 2.70 | 1.25 |
|  |  | 70-74 years | 2019 | 1.96 | 2.74 | 1.36 |
|  |  | 75-79 years | 2019 | 3.17 | 4.44 | 2.18 |
|  |  | 80-84 years | 2019 | 4.66 | 6.53 | 3.18 |
|  |  | 85+ years | 2019 | 7.62 | 10.09 | 5.36 |
| Kuwait | High-income | <5 years | 2019 | 0.01 | 0.02 | 0.01 |
|  |  | 5-9 years | 2019 | 0.03 | 0.04 | 0.02 |
|  |  | 10-14 years | 2019 | 0.10 | 0.14 | 0.07 |
|  |  | 15-19 years | 2019 | 0.12 | 0.17 | 0.08 |
|  |  | 20-24 years | 2019 | 0.08 | 0.12 | 0.05 |
|  |  | 25-29 years | 2019 | 0.10 | 0.14 | 0.06 |
|  |  | 30-34 years | 2019 | 0.11 | 0.16 | 0.07 |
|  |  | 35-39 years | 2019 | 0.13 | 0.19 | 0.08 |
|  |  | 40-44 years | 2019 | 0.20 | 0.30 | 0.12 |
|  |  | 45-49 years | 2019 | 0.25 | 0.38 | 0.15 |
|  |  | 50-54 years | 2019 | 0.30 | 0.46 | 0.19 |
|  |  | 55-59 years | 2019 | 0.46 | 0.67 | 0.30 |
|  |  | 60-64 years | 2019 | 0.73 | 1.05 | 0.49 |
|  |  | 65-69 years | 2019 | 1.23 | 1.69 | 0.86 |
|  |  | 70-74 years | 2019 | 1.88 | 2.63 | 1.31 |
|  |  | 75-79 years | 2019 | 2.60 | 3.60 | 1.88 |
|  |  | 80-84 years | 2019 | 5.62 | 7.79 | 3.89 |
|  |  | 85+ years | 2019 | 10.56 | 14.52 | 7.00 |
| Lebanon | Middle-income | <5 years | 2019 | 0.04 | 0.09 | 0.01 |
|  |  | 5-9 years | 2019 | 0.06 | 0.13 | 0.02 |
|  |  | 10-14 years | 2019 | 0.09 | 0.19 | 0.04 |
|  |  | 15-19 years | 2019 | 0.21 | 0.39 | 0.09 |
|  |  | 20-24 years | 2019 | 0.22 | 0.43 | 0.09 |
|  |  | 25-29 years | 2019 | 0.25 | 0.45 | 0.11 |
|  |  | 30-34 years | 2019 | 0.29 | 0.51 | 0.12 |
|  |  | 35-39 years | 2019 | 0.37 | 0.67 | 0.15 |
|  |  | 40-44 years | 2019 | 0.57 | 1.08 | 0.23 |
|  |  | 45-49 years | 2019 | 0.65 | 1.16 | 0.26 |
|  |  | 50-54 years | 2019 | 0.83 | 1.49 | 0.34 |
|  |  | 55-59 years | 2019 | 1.32 | 2.34 | 0.52 |
|  |  | 60-64 years | 2019 | 1.60 | 2.75 | 0.66 |
|  |  | 65-69 years | 2019 | 2.79 | 4.67 | 1.16 |
|  |  | 70-74 years | 2019 | 3.78 | 6.08 | 1.56 |
|  |  | 75-79 years | 2019 | 5.94 | 9.61 | 2.50 |
|  |  | 80-84 years | 2019 | 8.10 | 13.22 | 3.43 |
|  |  | 85+ years | 2019 | 14.16 | 22.48 | 5.88 |
| Libya | Middle-income | <5 years | 2019 | 0.85 | 1.60 | 0.34 |
|  |  | 5-9 years | 2019 | 0.27 | 0.50 | 0.13 |
|  |  | 10-14 years | 2019 | 0.21 | 0.38 | 0.11 |
|  |  | 15-19 years | 2019 | 0.28 | 0.45 | 0.16 |
|  |  | 20-24 years | 2019 | 0.28 | 0.50 | 0.14 |
|  |  | 25-29 years | 2019 | 0.35 | 0.65 | 0.17 |
|  |  | 30-34 years | 2019 | 0.35 | 0.65 | 0.16 |
|  |  | 35-39 years | 2019 | 0.43 | 0.79 | 0.20 |
|  |  | 40-44 years | 2019 | 0.63 | 1.14 | 0.29 |
|  |  | 45-49 years | 2019 | 0.78 | 1.36 | 0.38 |
|  |  | 50-54 years | 2019 | 0.94 | 1.62 | 0.50 |
|  |  | 55-59 years | 2019 | 1.46 | 2.44 | 0.83 |
|  |  | 60-64 years | 2019 | 2.08 | 3.18 | 1.18 |
|  |  | 65-69 years | 2019 | 3.58 | 5.41 | 2.23 |
|  |  | 70-74 years | 2019 | 4.29 | 6.37 | 2.78 |
|  |  | 75-79 years | 2019 | 7.04 | 10.44 | 4.72 |
|  |  | 80-84 years | 2019 | 7.58 | 11.12 | 4.99 |
|  |  | 85+ years | 2019 | 13.37 | 18.60 | 9.12 |
| Morocco | Middle-income | <5 years | 2019 | 0.16 | 0.32 | 0.06 |
|  |  | 5-9 years | 2019 | 0.20 | 0.32 | 0.10 |
|  |  | 10-14 years | 2019 | 0.26 | 0.43 | 0.13 |
|  |  | 15-19 years | 2019 | 0.29 | 0.48 | 0.16 |
|  |  | 20-24 years | 2019 | 0.33 | 0.59 | 0.17 |
|  |  | 25-29 years | 2019 | 0.44 | 0.80 | 0.23 |
|  |  | 30-34 years | 2019 | 0.58 | 1.05 | 0.29 |
|  |  | 35-39 years | 2019 | 0.77 | 1.35 | 0.40 |
|  |  | 40-44 years | 2019 | 1.44 | 2.45 | 0.80 |
|  |  | 45-49 years | 2019 | 1.88 | 3.02 | 1.08 |
|  |  | 50-54 years | 2019 | 2.53 | 4.01 | 1.54 |
|  |  | 55-59 years | 2019 | 4.05 | 6.08 | 2.55 |
|  |  | 60-64 years | 2019 | 4.87 | 6.96 | 3.18 |
|  |  | 65-69 years | 2019 | 7.76 | 11.12 | 5.09 |
|  |  | 70-74 years | 2019 | 10.14 | 14.60 | 6.84 |
|  |  | 75-79 years | 2019 | 15.72 | 22.62 | 10.98 |
|  |  | 80-84 years | 2019 | 21.18 | 33.67 | 13.83 |
|  |  | 85+ years | 2019 | 34.15 | 56.53 | 23.56 |
| Oman | High-income | <5 years | 2019 | 0.02 | 0.03 | 0.01 |
|  |  | 5-9 years | 2019 | 0.02 | 0.03 | 0.02 |
|  |  | 10-14 years | 2019 | 0.03 | 0.04 | 0.02 |
|  |  | 15-19 years | 2019 | 0.04 | 0.06 | 0.02 |
|  |  | 20-24 years | 2019 | 0.03 | 0.06 | 0.02 |
|  |  | 25-29 years | 2019 | 0.04 | 0.07 | 0.02 |
|  |  | 30-34 years | 2019 | 0.05 | 0.10 | 0.03 |
|  |  | 35-39 years | 2019 | 0.06 | 0.11 | 0.03 |
|  |  | 40-44 years | 2019 | 0.08 | 0.15 | 0.04 |
|  |  | 45-49 years | 2019 | 0.12 | 0.21 | 0.06 |
|  |  | 50-54 years | 2019 | 0.24 | 0.41 | 0.15 |
|  |  | 55-59 years | 2019 | 0.56 | 0.89 | 0.36 |
|  |  | 60-64 years | 2019 | 1.09 | 1.61 | 0.72 |
|  |  | 65-69 years | 2019 | 2.38 | 3.37 | 1.67 |
|  |  | 70-74 years | 2019 | 3.45 | 4.65 | 2.45 |
|  |  | 75-79 years | 2019 | 5.94 | 8.28 | 4.00 |
|  |  | 80-84 years | 2019 | 8.76 | 12.41 | 5.41 |
|  |  | 85+ years | 2019 | 14.98 | 19.92 | 10.28 |
| Pakistan | Low-income | <5 years | 2019 | 0.89 | 1.41 | 0.45 |
|  |  | 5-9 years | 2019 | 1.06 | 1.54 | 0.63 |
|  |  | 10-14 years | 2019 | 1.82 | 2.64 | 1.18 |
|  |  | 15-19 years | 2019 | 4.13 | 5.59 | 2.95 |
|  |  | 20-24 years | 2019 | 5.67 | 7.95 | 3.87 |
|  |  | 25-29 years | 2019 | 6.83 | 9.39 | 4.50 |
|  |  | 30-34 years | 2019 | 8.07 | 11.35 | 4.97 |
|  |  | 35-39 years | 2019 | 10.80 | 16.16 | 6.63 |
|  |  | 40-44 years | 2019 | 15.15 | 22.55 | 8.99 |
|  |  | 45-49 years | 2019 | 15.11 | 22.84 | 8.84 |
|  |  | 50-54 years | 2019 | 21.49 | 32.31 | 13.68 |
|  |  | 55-59 years | 2019 | 28.53 | 41.07 | 18.20 |
|  |  | 60-64 years | 2019 | 34.40 | 47.28 | 23.78 |
|  |  | 65-69 years | 2019 | 49.05 | 65.82 | 35.29 |
|  |  | 70-74 years | 2019 | 67.79 | 88.53 | 49.46 |
|  |  | 75-79 years | 2019 | 99.74 | 131.06 | 72.88 |
|  |  | 80-84 years | 2019 | 144.52 | 191.81 | 105.38 |
|  |  | 85+ years | 2019 | 234.54 | 297.33 | 177.17 |
| Occupied Palestinian territory | Middle-income | <5 years | 2019 | 0.030 | 0.053 | 0.015 |
|  |  | 5-9 years | 2019 | 0.019 | 0.032 | 0.011 |
|  |  | 10-14 years | 2019 | 0.025 | 0.043 | 0.013 |
|  |  | 15-19 years | 2019 | 0.049 | 0.076 | 0.030 |
|  |  | 20-24 years | 2019 | 0.086 | 0.133 | 0.053 |
|  |  | 25-29 years | 2019 | 0.128 | 0.189 | 0.083 |
|  |  | 30-34 years | 2019 | 0.158 | 0.236 | 0.102 |
|  |  | 35-39 years | 2019 | 0.224 | 0.319 | 0.147 |
|  |  | 40-44 years | 2019 | 0.427 | 0.607 | 0.289 |
|  |  | 45-49 years | 2019 | 0.489 | 0.670 | 0.341 |
|  |  | 50-54 years | 2019 | 0.605 | 0.856 | 0.414 |
|  |  | 55-59 years | 2019 | 1.043 | 1.431 | 0.719 |
|  |  | 60-64 years | 2019 | 1.405 | 1.917 | 1.014 |
|  |  | 65-69 years | 2019 | 2.359 | 3.181 | 1.641 |
|  |  | 70-74 years | 2019 | 3.170 | 4.242 | 2.275 |
|  |  | 75-79 years | 2019 | 5.906 | 7.932 | 3.984 |
|  |  | 80-84 years | 2019 | 8.262 | 10.778 | 6.028 |
|  |  | 85+ years | 2019 | 17.774 | 22.490 | 13.215 |
| Qatar | High-income | <5 years | 2019 | 0.03 | 0.05 | 0.01 |
|  |  | 5-9 years | 2019 | 0.05 | 0.09 | 0.02 |
|  |  | 10-14 years | 2019 | 0.04 | 0.07 | 0.02 |
|  |  | 15-19 years | 2019 | 0.09 | 0.16 | 0.05 |
|  |  | 20-24 years | 2019 | 0.11 | 0.21 | 0.05 |
|  |  | 25-29 years | 2019 | 0.08 | 0.14 | 0.04 |
|  |  | 30-34 years | 2019 | 0.11 | 0.18 | 0.06 |
|  |  | 35-39 years | 2019 | 0.17 | 0.28 | 0.10 |
|  |  | 40-44 years | 2019 | 0.23 | 0.38 | 0.14 |
|  |  | 45-49 years | 2019 | 0.34 | 0.52 | 0.21 |
|  |  | 50-54 years | 2019 | 0.47 | 0.76 | 0.28 |
|  |  | 55-59 years | 2019 | 0.84 | 1.34 | 0.49 |
|  |  | 60-64 years | 2019 | 0.67 | 1.06 | 0.41 |
|  |  | 65-69 years | 2019 | 1.34 | 2.06 | 0.84 |
|  |  | 70-74 years | 2019 | 2.74 | 4.07 | 1.84 |
|  |  | 75-79 years | 2019 | 8.01 | 11.54 | 5.56 |
|  |  | 80-84 years | 2019 | 20.94 | 28.78 | 14.57 |
|  |  | 85+ years | 2019 | 20.80 | 28.10 | 15.30 |
| Saudi Arabia | High-income | <5 years | 2019 | 0.01 | 0.03 | 0.00 |
|  |  | 5-9 years | 2019 | 0.02 | 0.03 | 0.01 |
|  |  | 10-14 years | 2019 | 0.06 | 0.11 | 0.03 |
|  |  | 15-19 years | 2019 | 0.15 | 0.24 | 0.09 |
|  |  | 20-24 years | 2019 | 0.20 | 0.34 | 0.11 |
|  |  | 25-29 years | 2019 | 0.25 | 0.43 | 0.13 |
|  |  | 30-34 years | 2019 | 0.39 | 0.66 | 0.21 |
|  |  | 35-39 years | 2019 | 0.75 | 1.25 | 0.40 |
|  |  | 40-44 years | 2019 | 1.08 | 1.82 | 0.62 |
|  |  | 45-49 years | 2019 | 1.20 | 1.89 | 0.68 |
|  |  | 50-54 years | 2019 | 1.36 | 2.05 | 0.82 |
|  |  | 55-59 years | 2019 | 2.03 | 2.93 | 1.27 |
|  |  | 60-64 years | 2019 | 2.01 | 2.90 | 1.30 |
|  |  | 65-69 years | 2019 | 3.08 | 4.51 | 1.96 |
|  |  | 70-74 years | 2019 | 3.47 | 4.96 | 2.26 |
|  |  | 75-79 years | 2019 | 4.79 | 6.73 | 3.20 |
|  |  | 80-84 years | 2019 | 6.04 | 8.58 | 3.70 |
|  |  | 85+ years | 2019 | 10.56 | 14.45 | 6.79 |
| Somalia | Low-income | <5 years | 2019 | 0.67 | 1.42 | 0.25 |
|  |  | 5-9 years | 2019 | 0.77 | 1.34 | 0.39 |
|  |  | 10-14 years | 2019 | 1.31 | 2.42 | 0.65 |
|  |  | 15-19 years | 2019 | 1.94 | 3.85 | 0.90 |
|  |  | 20-24 years | 2019 | 1.57 | 3.21 | 0.74 |
|  |  | 25-29 years | 2019 | 1.21 | 2.34 | 0.54 |
|  |  | 30-34 years | 2019 | 1.21 | 2.37 | 0.55 |
|  |  | 35-39 years | 2019 | 1.55 | 3.14 | 0.65 |
|  |  | 40-44 years | 2019 | 2.11 | 4.22 | 0.92 |
|  |  | 45-49 years | 2019 | 2.35 | 4.82 | 0.97 |
|  |  | 50-54 years | 2019 | 4.40 | 8.79 | 1.86 |
|  |  | 55-59 years | 2019 | 7.59 | 15.23 | 3.48 |
|  |  | 60-64 years | 2019 | 11.94 | 25.58 | 5.39 |
|  |  | 65-69 years | 2019 | 21.15 | 43.85 | 9.87 |
|  |  | 70-74 years | 2019 | 27.04 | 51.63 | 13.07 |
|  |  | 75-79 years | 2019 | 33.41 | 63.47 | 16.57 |
|  |  | 80-84 years | 2019 | 41.78 | 81.86 | 20.34 |
|  |  | 85+ years | 2019 | 63.92 | 122.39 | 32.43 |
| Sudan | Low-income | <5 years | 2019 | 0.88 | 1.93 | 0.37 |
|  |  | 5-9 years | 2019 | 0.47 | 0.88 | 0.22 |
|  |  | 10-14 years | 2019 | 0.47 | 0.90 | 0.23 |
|  |  | 15-19 years | 2019 | 0.44 | 0.94 | 0.19 |
|  |  | 20-24 years | 2019 | 0.54 | 1.08 | 0.22 |
|  |  | 25-29 years | 2019 | 0.72 | 1.37 | 0.30 |
|  |  | 30-34 years | 2019 | 0.91 | 1.62 | 0.39 |
|  |  | 35-39 years | 2019 | 1.12 | 2.01 | 0.53 |
|  |  | 40-44 years | 2019 | 1.91 | 3.31 | 0.94 |
|  |  | 45-49 years | 2019 | 2.33 | 4.06 | 1.23 |
|  |  | 50-54 years | 2019 | 3.19 | 5.25 | 1.81 |
|  |  | 55-59 years | 2019 | 4.86 | 8.16 | 2.74 |
|  |  | 60-64 years | 2019 | 5.90 | 10.35 | 3.39 |
|  |  | 65-69 years | 2019 | 9.12 | 15.60 | 5.27 |
|  |  | 70-74 years | 2019 | 12.04 | 20.90 | 6.97 |
|  |  | 75-79 years | 2019 | 17.92 | 30.88 | 10.95 |
|  |  | 80-84 years | 2019 | 23.05 | 41.02 | 13.78 |
|  |  | 85+ years | 2019 | 33.82 | 58.77 | 21.34 |
| Syrian Arab Republic | Middle-income | <5 years | 2019 | 0.55 | 0.91 | 0.31 |
|  |  | 5-9 years | 2019 | 0.48 | 0.85 | 0.26 |
|  |  | 10-14 years | 2019 | 0.49 | 0.75 | 0.28 |
|  |  | 15-19 years | 2019 | 0.33 | 0.51 | 0.20 |
|  |  | 20-24 years | 2019 | 0.36 | 0.57 | 0.21 |
|  |  | 25-29 years | 2019 | 0.47 | 0.78 | 0.25 |
|  |  | 30-34 years | 2019 | 0.50 | 0.82 | 0.29 |
|  |  | 35-39 years | 2019 | 0.65 | 1.05 | 0.38 |
|  |  | 40-44 years | 2019 | 1.06 | 1.70 | 0.61 |
|  |  | 45-49 years | 2019 | 1.21 | 1.97 | 0.69 |
|  |  | 50-54 years | 2019 | 1.87 | 3.08 | 1.09 |
|  |  | 55-59 years | 2019 | 3.25 | 5.14 | 1.92 |
|  |  | 60-64 years | 2019 | 3.70 | 5.73 | 2.24 |
|  |  | 65-69 years | 2019 | 5.51 | 8.09 | 3.44 |
|  |  | 70-74 years | 2019 | 7.65 | 11.28 | 4.87 |
|  |  | 75-79 years | 2019 | 10.30 | 14.57 | 6.68 |
|  |  | 80-84 years | 2019 | 11.17 | 15.78 | 7.49 |
|  |  | 85+ years | 2019 | 21.66 | 29.54 | 14.83 |
| Tunisia | Middle-income | <5 years | 2019 | 0.08 | 0.15 | 0.03 |
|  |  | 5-9 years | 2019 | 0.08 | 0.14 | 0.05 |
|  |  | 10-14 years | 2019 | 0.11 | 0.18 | 0.06 |
|  |  | 15-19 years | 2019 | 0.11 | 0.19 | 0.06 |
|  |  | 20-24 years | 2019 | 0.15 | 0.26 | 0.08 |
|  |  | 25-29 years | 2019 | 0.21 | 0.35 | 0.12 |
|  |  | 30-34 years | 2019 | 0.30 | 0.49 | 0.16 |
|  |  | 35-39 years | 2019 | 0.39 | 0.63 | 0.22 |
|  |  | 40-44 years | 2019 | 0.64 | 1.03 | 0.36 |
|  |  | 45-49 years | 2019 | 0.75 | 1.23 | 0.40 |
|  |  | 50-54 years | 2019 | 0.96 | 1.57 | 0.56 |
|  |  | 55-59 years | 2019 | 1.72 | 2.82 | 0.92 |
|  |  | 60-64 years | 2019 | 2.08 | 3.31 | 1.17 |
|  |  | 65-69 years | 2019 | 3.60 | 5.77 | 2.09 |
|  |  | 70-74 years | 2019 | 4.91 | 7.40 | 2.99 |
|  |  | 75-79 years | 2019 | 8.04 | 12.01 | 4.86 |
|  |  | 80-84 years | 2019 | 11.71 | 17.52 | 6.76 |
|  |  | 85+ years | 2019 | 19.69 | 28.15 | 11.72 |
| United Arab Emirates | High-income | <5 years | 2019 | 0.62 | 1.16 | 0.29 |
|  |  | 5-9 years | 2019 | 0.53 | 0.90 | 0.30 |
|  |  | 10-14 years | 2019 | 0.58 | 1.00 | 0.30 |
|  |  | 15-19 years | 2019 | 0.41 | 0.82 | 0.15 |
|  |  | 20-24 years | 2019 | 0.71 | 1.34 | 0.29 |
|  |  | 25-29 years | 2019 | 0.90 | 1.65 | 0.41 |
|  |  | 30-34 years | 2019 | 1.20 | 2.22 | 0.52 |
|  |  | 35-39 years | 2019 | 1.72 | 3.26 | 0.82 |
|  |  | 40-44 years | 2019 | 3.30 | 5.85 | 1.61 |
|  |  | 45-49 years | 2019 | 3.97 | 7.22 | 2.09 |
|  |  | 50-54 years | 2019 | 5.31 | 9.15 | 2.87 |
|  |  | 55-59 years | 2019 | 8.28 | 14.06 | 4.48 |
|  |  | 60-64 years | 2019 | 9.80 | 16.27 | 5.67 |
|  |  | 65-69 years | 2019 | 14.74 | 24.48 | 8.46 |
|  |  | 70-74 years | 2019 | 18.23 | 29.67 | 10.85 |
|  |  | 75-79 years | 2019 | 26.16 | 42.92 | 16.22 |
|  |  | 80-84 years | 2019 | 32.99 | 52.99 | 19.89 |
|  |  | 85+ years | 2019 | 47.92 | 75.29 | 29.61 |
| Yemen | Low-income | <5 years | 2019 | 0.07 | 0.14 | 0.03 |
|  |  | 5-9 years | 2019 | 0.09 | 0.15 | 0.04 |
|  |  | 10-14 years | 2019 | 0.27 | 0.47 | 0.13 |
|  |  | 15-19 years | 2019 | 0.08 | 0.14 | 0.04 |
|  |  | 20-24 years | 2019 | 0.20 | 0.41 | 0.08 |
|  |  | 25-29 years | 2019 | 0.38 | 0.74 | 0.17 |
|  |  | 30-34 years | 2019 | 0.50 | 1.06 | 0.19 |
|  |  | 35-39 years | 2019 | 0.67 | 1.36 | 0.29 |
|  |  | 40-44 years | 2019 | 1.14 | 2.22 | 0.54 |
|  |  | 45-49 years | 2019 | 1.80 | 3.23 | 0.93 |
|  |  | 50-54 years | 2019 | 3.36 | 5.89 | 1.74 |
|  |  | 55-59 years | 2019 | 8.92 | 16.38 | 4.50 |
|  |  | 60-64 years | 2019 | 10.61 | 18.68 | 5.34 |
|  |  | 65-69 years | 2019 | 21.11 | 37.62 | 10.02 |
|  |  | 70-74 years | 2019 | 18.43 | 32.92 | 8.71 |
|  |  | 75-79 years | 2019 | 26.29 | 46.15 | 13.31 |
|  |  | 80-84 years | 2019 | 35.29 | 54.01 | 22.62 |
|  |  | 85+ years | 2019 | 31.40 | 46.15 | 21.60 |

**Supplementary Table 1B. Prevalence rate estimates with uncertainty intervals from IHME GBD 2019**

| Country | Group | Age | year | Prevalence rate (Per 100,000) | Upper value | Lower value |
| --- | --- | --- | --- | --- | --- | --- |
| Afghanistan | Low-income | <5 years | 2019 | 36.92 | 61.13 | 18.78 |
|  |  | 5-9 years | 2019 | 210.55 | 321.60 | 129.40 |
|  |  | 10-14 years | 2019 | 405.42 | 639.39 | 232.97 |
|  |  | 15-19 years | 2019 | 589.37 | 952.17 | 356.78 |
|  |  | 20-24 years | 2019 | 734.89 | 1023.89 | 494.49 |
|  |  | 25-29 years | 2019 | 815.22 | 1131.47 | 549.71 |
|  |  | 30-34 years | 2019 | 840.79 | 1162.69 | 591.83 |
|  |  | 35-39 years | 2019 | 826.34 | 1096.28 | 624.35 |
|  |  | 40-44 years | 2019 | 774.47 | 979.53 | 592.47 |
|  |  | 45-49 years | 2019 | 671.25 | 849.11 | 517.23 |
|  |  | 50-54 years | 2019 | 533.47 | 683.90 | 410.52 |
|  |  | 55-59 years | 2019 | 417.65 | 544.42 | 318.11 |
|  |  | 60-64 years | 2019 | 326.27 | 430.29 | 248.23 |
|  |  | 65-69 years | 2019 | 254.51 | 338.09 | 190.14 |
|  |  | 70-74 years | 2019 | 197.96 | 264.05 | 147.53 |
|  |  | 75-79 years | 2019 | 153.65 | 209.14 | 113.09 |
|  |  | 80-84 years | 2019 | 119.01 | 164.41 | 86.03 |
|  |  | 85+ years | 2019 | 85.87 | 121.22 | 60.45 |
| Bahrain | High-income | <5 years | 2019 | 2.24 | 3.64 | 1.11 |
|  |  | 5-9 years | 2019 | 5.95 | 9.39 | 3.35 |
|  |  | 10-14 years | 2019 | 8.76 | 13.88 | 5.20 |
|  |  | 15-19 years | 2019 | 11.04 | 16.58 | 7.18 |
|  |  | 20-24 years | 2019 | 12.90 | 18.56 | 8.64 |
|  |  | 25-29 years | 2019 | 14.69 | 21.26 | 9.80 |
|  |  | 30-34 years | 2019 | 16.63 | 24.46 | 10.78 |
|  |  | 35-39 years | 2019 | 19.62 | 27.57 | 13.30 |
|  |  | 40-44 years | 2019 | 25.34 | 35.99 | 17.14 |
|  |  | 45-49 years | 2019 | 34.30 | 46.57 | 23.76 |
|  |  | 50-54 years | 2019 | 46.31 | 64.72 | 32.13 |
|  |  | 55-59 years | 2019 | 64.99 | 88.62 | 45.73 |
|  |  | 60-64 years | 2019 | 90.10 | 128.31 | 60.67 |
|  |  | 65-69 years | 2019 | 120.93 | 162.34 | 86.27 |
|  |  | 70-74 years | 2019 | 162.92 | 214.81 | 112.55 |
|  |  | 75-79 years | 2019 | 217.34 | 286.48 | 153.01 |
|  |  | 80-84 years | 2019 | 277.26 | 370.79 | 194.70 |
|  |  | 85+ years | 2019 | 331.86 | 437.45 | 243.80 |
| Djibouti | Low-income | <5 years | 2019 | 70.09 | 114.55 | 36.71 |
|  |  | 5-9 years | 2019 | 452.61 | 687.58 | 276.54 |
|  |  | 10-14 years | 2019 | 1094.24 | 1742.51 | 641.61 |
|  |  | 15-19 years | 2019 | 1754.07 | 2572.24 | 1126.48 |
|  |  | 20-24 years | 2019 | 1950.40 | 2651.63 | 1394.74 |
|  |  | 25-29 years | 2019 | 1967.98 | 2600.61 | 1418.85 |
|  |  | 30-34 years | 2019 | 1919.14 | 2514.47 | 1401.58 |
|  |  | 35-39 years | 2019 | 1803.80 | 2326.76 | 1384.12 |
|  |  | 40-44 years | 2019 | 1633.41 | 2096.09 | 1268.95 |
|  |  | 45-49 years | 2019 | 1402.20 | 1801.01 | 1095.46 |
|  |  | 50-54 years | 2019 | 1134.23 | 1460.04 | 875.42 |
|  |  | 55-59 years | 2019 | 905.57 | 1205.71 | 684.96 |
|  |  | 60-64 years | 2019 | 723.23 | 979.87 | 541.10 |
|  |  | 65-69 years | 2019 | 577.54 | 781.66 | 428.28 |
|  |  | 70-74 years | 2019 | 458.40 | 611.76 | 339.92 |
|  |  | 75-79 years | 2019 | 366.79 | 500.20 | 272.40 |
|  |  | 80-84 years | 2019 | 293.19 | 399.44 | 213.67 |
|  |  | 85+ years | 2019 | 220.35 | 306.92 | 157.14 |
| Egypt | Middle-income | <5 years | 2019 | 38.97 | 63.29 | 20.16 |
|  |  | 5-9 years | 2019 | 219.75 | 338.22 | 135.16 |
|  |  | 10-14 years | 2019 | 428.34 | 686.87 | 251.72 |
|  |  | 15-19 years | 2019 | 622.00 | 938.82 | 380.07 |
|  |  | 20-24 years | 2019 | 772.03 | 1059.68 | 528.43 |
|  |  | 25-29 years | 2019 | 866.72 | 1165.67 | 606.25 |
|  |  | 30-34 years | 2019 | 913.68 | 1213.87 | 655.86 |
|  |  | 35-39 years | 2019 | 923.34 | 1193.28 | 703.70 |
|  |  | 40-44 years | 2019 | 893.96 | 1136.50 | 694.63 |
|  |  | 45-49 years | 2019 | 799.11 | 1009.31 | 617.65 |
|  |  | 50-54 years | 2019 | 658.22 | 841.54 | 510.99 |
|  |  | 55-59 years | 2019 | 526.78 | 677.51 | 402.66 |
|  |  | 60-64 years | 2019 | 415.90 | 546.00 | 314.79 |
|  |  | 65-69 years | 2019 | 328.23 | 429.95 | 244.97 |
|  |  | 70-74 years | 2019 | 257.97 | 341.51 | 192.59 |
|  |  | 75-79 years | 2019 | 203.92 | 273.45 | 152.24 |
|  |  | 80-84 years | 2019 | 161.10 | 221.57 | 116.75 |
|  |  | 85+ years | 2019 | 116.32 | 165.13 | 80.50 |
| Iran, Islamic Republic of | Middle-income | <5 years | 2019 | 31.48 | 52.24 | 16.18 |
|  |  | 5-9 years | 2019 | 186.21 | 282.53 | 113.65 |
|  |  | 10-14 years | 2019 | 396.18 | 625.93 | 231.01 |
|  |  | 15-19 years | 2019 | 585.80 | 865.92 | 369.42 |
|  |  | 20-24 years | 2019 | 699.29 | 956.98 | 491.15 |
|  |  | 25-29 years | 2019 | 765.84 | 1031.48 | 543.67 |
|  |  | 30-34 years | 2019 | 797.87 | 1060.37 | 594.79 |
|  |  | 35-39 years | 2019 | 801.93 | 1034.67 | 615.11 |
|  |  | 40-44 years | 2019 | 779.39 | 985.94 | 609.51 |
|  |  | 45-49 years | 2019 | 706.94 | 888.77 | 554.67 |
|  |  | 50-54 years | 2019 | 599.52 | 758.63 | 470.80 |
|  |  | 55-59 years | 2019 | 496.21 | 639.52 | 386.80 |
|  |  | 60-64 years | 2019 | 407.73 | 523.92 | 318.02 |
|  |  | 65-69 years | 2019 | 332.35 | 427.36 | 257.66 |
|  |  | 70-74 years | 2019 | 269.72 | 350.46 | 207.22 |
|  |  | 75-79 years | 2019 | 215.81 | 285.53 | 164.20 |
|  |  | 80-84 years | 2019 | 173.09 | 233.49 | 129.49 |
|  |  | 85+ years | 2019 | 129.30 | 177.58 | 95.45 |
| Iraq | Middle-income | <5 years | 2019 | 37.90 | 62.28 | 19.43 |
|  |  | 5-9 years | 2019 | 216.06 | 324.56 | 129.76 |
|  |  | 10-14 years | 2019 | 417.62 | 659.04 | 243.65 |
|  |  | 15-19 years | 2019 | 595.82 | 884.64 | 366.29 |
|  |  | 20-24 years | 2019 | 723.42 | 996.67 | 503.10 |
|  |  | 25-29 years | 2019 | 799.58 | 1077.95 | 552.41 |
|  |  | 30-34 years | 2019 | 836.13 | 1125.46 | 605.17 |
|  |  | 35-39 years | 2019 | 840.32 | 1088.84 | 643.09 |
|  |  | 40-44 years | 2019 | 811.83 | 1042.24 | 621.48 |
|  |  | 45-49 years | 2019 | 726.65 | 920.88 | 561.62 |
|  |  | 50-54 years | 2019 | 601.16 | 766.41 | 461.10 |
|  |  | 55-59 years | 2019 | 487.09 | 631.90 | 373.91 |
|  |  | 60-64 years | 2019 | 392.07 | 517.38 | 298.74 |
|  |  | 65-69 years | 2019 | 314.02 | 411.80 | 235.90 |
|  |  | 70-74 years | 2019 | 250.74 | 332.01 | 185.72 |
|  |  | 75-79 years | 2019 | 200.69 | 270.24 | 148.71 |
|  |  | 80-84 years | 2019 | 161.51 | 222.24 | 116.66 |
|  |  | 85+ years | 2019 | 120.08 | 169.94 | 83.79 |
| Jordan | Middle-income | <5 years | 2019 | 2.01 | 3.23 | 0.97 |
|  |  | 5-9 years | 2019 | 5.25 | 8.27 | 2.85 |
|  |  | 10-14 years | 2019 | 7.57 | 12.14 | 4.33 |
|  |  | 15-19 years | 2019 | 9.39 | 14.34 | 5.90 |
|  |  | 20-24 years | 2019 | 10.88 | 16.09 | 7.12 |
|  |  | 25-29 years | 2019 | 12.31 | 18.39 | 8.07 |
|  |  | 30-34 years | 2019 | 13.97 | 20.77 | 8.77 |
|  |  | 35-39 years | 2019 | 17.06 | 24.87 | 11.07 |
|  |  | 40-44 years | 2019 | 21.55 | 31.30 | 13.81 |
|  |  | 45-49 years | 2019 | 27.69 | 38.88 | 19.09 |
|  |  | 50-54 years | 2019 | 35.30 | 49.13 | 24.45 |
|  |  | 55-59 years | 2019 | 43.71 | 59.93 | 30.91 |
|  |  | 60-64 years | 2019 | 52.64 | 72.35 | 35.95 |
|  |  | 65-69 years | 2019 | 62.25 | 84.12 | 44.14 |
|  |  | 70-74 years | 2019 | 73.17 | 96.22 | 50.74 |
|  |  | 75-79 years | 2019 | 86.86 | 113.48 | 61.03 |
|  |  | 80-84 years | 2019 | 103.81 | 138.02 | 72.50 |
|  |  | 85+ years | 2019 | 132.86 | 180.79 | 94.17 |
| Kuwait | High-income | <5 years | 2019 | 2.08 | 3.39 | 1.03 |
|  |  | 5-9 years | 2019 | 5.57 | 8.77 | 3.19 |
|  |  | 10-14 years | 2019 | 8.14 | 12.85 | 4.77 |
|  |  | 15-19 years | 2019 | 10.24 | 15.61 | 6.54 |
|  |  | 20-24 years | 2019 | 12.12 | 17.54 | 7.95 |
|  |  | 25-29 years | 2019 | 14.12 | 20.98 | 9.07 |
|  |  | 30-34 years | 2019 | 16.55 | 24.05 | 10.39 |
|  |  | 35-39 years | 2019 | 20.14 | 28.63 | 13.24 |
|  |  | 40-44 years | 2019 | 25.51 | 36.24 | 16.58 |
|  |  | 45-49 years | 2019 | 33.22 | 45.62 | 22.29 |
|  |  | 50-54 years | 2019 | 43.04 | 61.80 | 28.92 |
|  |  | 55-59 years | 2019 | 56.63 | 78.39 | 39.66 |
|  |  | 60-64 years | 2019 | 73.51 | 102.24 | 50.00 |
|  |  | 65-69 years | 2019 | 94.10 | 130.36 | 65.68 |
|  |  | 70-74 years | 2019 | 117.21 | 154.99 | 80.95 |
|  |  | 75-79 years | 2019 | 149.50 | 191.69 | 108.28 |
|  |  | 80-84 years | 2019 | 186.84 | 243.69 | 135.43 |
|  |  | 85+ years | 2019 | 258.83 | 337.98 | 192.80 |
| Lebanon | Middle-income | <5 years | 2019 | 2.29 | 3.70 | 1.21 |
|  |  | 5-9 years | 2019 | 6.34 | 9.86 | 3.68 |
|  |  | 10-14 years | 2019 | 9.48 | 15.31 | 5.67 |
|  |  | 15-19 years | 2019 | 12.03 | 18.07 | 7.83 |
|  |  | 20-24 years | 2019 | 14.02 | 20.20 | 9.38 |
|  |  | 25-29 years | 2019 | 15.89 | 22.54 | 10.59 |
|  |  | 30-34 years | 2019 | 18.19 | 25.99 | 12.00 |
|  |  | 35-39 years | 2019 | 22.57 | 31.74 | 15.37 |
|  |  | 40-44 years | 2019 | 28.96 | 39.83 | 19.85 |
|  |  | 45-49 years | 2019 | 36.91 | 48.62 | 26.74 |
|  |  | 50-54 years | 2019 | 46.79 | 64.82 | 33.62 |
|  |  | 55-59 years | 2019 | 60.86 | 82.95 | 43.27 |
|  |  | 60-64 years | 2019 | 78.65 | 109.00 | 52.72 |
|  |  | 65-69 years | 2019 | 100.16 | 134.97 | 69.36 |
|  |  | 70-74 years | 2019 | 124.66 | 165.24 | 83.51 |
|  |  | 75-79 years | 2019 | 151.04 | 200.91 | 103.22 |
|  |  | 80-84 years | 2019 | 178.10 | 243.18 | 118.07 |
|  |  | 85+ years | 2019 | 213.56 | 293.33 | 151.45 |
| Libya | Middle-income | <5 years | 2019 | 38.60 | 64.15 | 19.51 |
|  |  | 5-9 years | 2019 | 217.79 | 336.79 | 129.81 |
|  |  | 10-14 years | 2019 | 420.02 | 655.00 | 245.72 |
|  |  | 15-19 years | 2019 | 595.05 | 889.86 | 372.70 |
|  |  | 20-24 years | 2019 | 719.52 | 985.73 | 499.91 |
|  |  | 25-29 years | 2019 | 796.62 | 1077.48 | 553.64 |
|  |  | 30-34 years | 2019 | 834.15 | 1125.19 | 606.27 |
|  |  | 35-39 years | 2019 | 841.93 | 1106.60 | 641.78 |
|  |  | 40-44 years | 2019 | 818.35 | 1062.92 | 636.59 |
|  |  | 45-49 years | 2019 | 742.16 | 946.64 | 580.72 |
|  |  | 50-54 years | 2019 | 626.37 | 810.38 | 484.11 |
|  |  | 55-59 years | 2019 | 515.14 | 673.54 | 399.75 |
|  |  | 60-64 years | 2019 | 419.34 | 553.83 | 321.46 |
|  |  | 65-69 years | 2019 | 338.31 | 445.00 | 256.59 |
|  |  | 70-74 years | 2019 | 269.04 | 355.41 | 205.47 |
|  |  | 75-79 years | 2019 | 213.03 | 287.17 | 160.52 |
|  |  | 80-84 years | 2019 | 169.57 | 233.29 | 124.39 |
|  |  | 85+ years | 2019 | 124.66 | 172.78 | 87.15 |
| Morocco | Middle-income | <5 years | 2019 | 38.50 | 63.91 | 19.99 |
|  |  | 5-9 years | 2019 | 217.02 | 335.04 | 132.15 |
|  |  | 10-14 years | 2019 | 418.61 | 666.39 | 245.14 |
|  |  | 15-19 years | 2019 | 599.57 | 901.73 | 377.46 |
|  |  | 20-24 years | 2019 | 735.16 | 1000.54 | 512.51 |
|  |  | 25-29 years | 2019 | 817.33 | 1120.36 | 561.13 |
|  |  | 30-34 years | 2019 | 855.37 | 1160.83 | 615.55 |
|  |  | 35-39 years | 2019 | 859.31 | 1121.39 | 650.32 |
|  |  | 40-44 years | 2019 | 828.76 | 1061.26 | 640.42 |
|  |  | 45-49 years | 2019 | 743.25 | 948.52 | 578.20 |
|  |  | 50-54 years | 2019 | 617.84 | 790.44 | 477.54 |
|  |  | 55-59 years | 2019 | 500.40 | 651.24 | 381.62 |
|  |  | 60-64 years | 2019 | 400.99 | 527.42 | 303.04 |
|  |  | 65-69 years | 2019 | 318.78 | 411.29 | 238.55 |
|  |  | 70-74 years | 2019 | 253.17 | 331.33 | 188.04 |
|  |  | 75-79 years | 2019 | 202.16 | 268.51 | 150.57 |
|  |  | 80-84 years | 2019 | 160.72 | 217.14 | 117.48 |
|  |  | 85+ years | 2019 | 116.47 | 162.01 | 83.03 |
| Oman | High-income | <5 years | 2019 | 2.25 | 3.75 | 1.16 |
|  |  | 5-9 years | 2019 | 5.98 | 9.43 | 3.47 |
|  |  | 10-14 years | 2019 | 8.63 | 14.03 | 5.06 |
|  |  | 15-19 years | 2019 | 10.74 | 16.61 | 6.74 |
|  |  | 20-24 years | 2019 | 11.97 | 17.44 | 7.68 |
|  |  | 25-29 years | 2019 | 13.27 | 19.83 | 8.51 |
|  |  | 30-34 years | 2019 | 15.15 | 22.65 | 9.42 |
|  |  | 35-39 years | 2019 | 18.76 | 27.04 | 12.02 |
|  |  | 40-44 years | 2019 | 24.45 | 35.21 | 15.75 |
|  |  | 45-49 years | 2019 | 31.98 | 44.69 | 21.58 |
|  |  | 50-54 years | 2019 | 42.35 | 60.39 | 29.00 |
|  |  | 55-59 years | 2019 | 57.56 | 79.27 | 39.33 |
|  |  | 60-64 years | 2019 | 78.71 | 110.63 | 52.08 |
|  |  | 65-69 years | 2019 | 108.29 | 146.15 | 73.71 |
|  |  | 70-74 years | 2019 | 141.60 | 187.35 | 96.72 |
|  |  | 75-79 years | 2019 | 178.48 | 232.12 | 125.12 |
|  |  | 80-84 years | 2019 | 218.22 | 291.35 | 151.90 |
|  |  | 85+ years | 2019 | 280.05 | 373.59 | 201.02 |
| Pakistan | Low-income | <5 years | 2019 | 30.71 | 50.76 | 15.84 |
|  |  | 5-9 years | 2019 | 193.51 | 297.21 | 117.02 |
|  |  | 10-14 years | 2019 | 463.30 | 739.03 | 266.95 |
|  |  | 15-19 years | 2019 | 827.17 | 1267.79 | 521.60 |
|  |  | 20-24 years | 2019 | 1183.36 | 1577.84 | 831.91 |
|  |  | 25-29 years | 2019 | 1383.14 | 1838.21 | 980.46 |
|  |  | 30-34 years | 2019 | 1429.66 | 1866.62 | 1040.50 |
|  |  | 35-39 years | 2019 | 1359.67 | 1741.50 | 1033.13 |
|  |  | 40-44 years | 2019 | 1208.42 | 1547.07 | 937.49 |
|  |  | 45-49 years | 2019 | 1014.84 | 1303.19 | 781.63 |
|  |  | 50-54 years | 2019 | 805.56 | 1041.19 | 609.02 |
|  |  | 55-59 years | 2019 | 634.46 | 840.12 | 477.01 |
|  |  | 60-64 years | 2019 | 503.55 | 668.43 | 378.02 |
|  |  | 65-69 years | 2019 | 399.51 | 529.21 | 300.92 |
|  |  | 70-74 years | 2019 | 316.11 | 425.05 | 233.57 |
|  |  | 75-79 years | 2019 | 251.33 | 343.68 | 185.88 |
|  |  | 80-84 years | 2019 | 199.57 | 276.53 | 145.42 |
|  |  | 85+ years | 2019 | 147.07 | 209.16 | 103.67 |
| Occupied Palestinian territory | Middle-income | <5 years | 2019 | 36.46 | 60.13 | 18.40 |
|  |  | 5-9 years | 2019 | 208.25 | 326.32 | 126.51 |
|  |  | 10-14 years | 2019 | 404.09 | 653.99 | 239.55 |
|  |  | 15-19 years | 2019 | 573.71 | 863.92 | 355.31 |
|  |  | 20-24 years | 2019 | 700.14 | 951.68 | 492.01 |
|  |  | 25-29 years | 2019 | 783.15 | 1054.50 | 554.06 |
|  |  | 30-34 years | 2019 | 831.51 | 1108.59 | 614.59 |
|  |  | 35-39 years | 2019 | 850.95 | 1102.59 | 652.03 |
|  |  | 40-44 years | 2019 | 836.41 | 1075.18 | 653.70 |
|  |  | 45-49 years | 2019 | 760.09 | 979.82 | 587.72 |
|  |  | 50-54 years | 2019 | 636.88 | 821.42 | 488.64 |
|  |  | 55-59 years | 2019 | 516.09 | 680.09 | 397.36 |
|  |  | 60-64 years | 2019 | 412.49 | 544.33 | 317.71 |
|  |  | 65-69 years | 2019 | 324.74 | 431.07 | 246.67 |
|  |  | 70-74 years | 2019 | 255.11 | 342.37 | 193.82 |
|  |  | 75-79 years | 2019 | 201.44 | 271.58 | 153.35 |
|  |  | 80-84 years | 2019 | 158.60 | 219.23 | 118.69 |
|  |  | 85+ years | 2019 | 116.18 | 163.16 | 84.12 |
| Qatar | High-income | <5 years | 2019 | 2.13 | 3.36 | 1.05 |
|  |  | 5-9 years | 2019 | 5.59 | 8.63 | 3.16 |
|  |  | 10-14 years | 2019 | 8.12 | 12.70 | 4.86 |
|  |  | 15-19 years | 2019 | 9.95 | 14.99 | 6.42 |
|  |  | 20-24 years | 2019 | 10.84 | 16.16 | 7.02 |
|  |  | 25-29 years | 2019 | 12.42 | 18.50 | 8.11 |
|  |  | 30-34 years | 2019 | 14.70 | 21.93 | 9.25 |
|  |  | 35-39 years | 2019 | 18.93 | 26.94 | 12.30 |
|  |  | 40-44 years | 2019 | 25.25 | 35.41 | 16.75 |
|  |  | 45-49 years | 2019 | 34.30 | 46.68 | 23.55 |
|  |  | 50-54 years | 2019 | 47.33 | 66.48 | 32.23 |
|  |  | 55-59 years | 2019 | 66.40 | 92.54 | 45.30 |
|  |  | 60-64 years | 2019 | 93.17 | 134.93 | 61.18 |
|  |  | 65-69 years | 2019 | 130.98 | 180.03 | 87.37 |
|  |  | 70-74 years | 2019 | 182.32 | 243.38 | 120.17 |
|  |  | 75-79 years | 2019 | 258.07 | 339.41 | 183.09 |
|  |  | 80-84 years | 2019 | 314.59 | 406.37 | 224.44 |
|  |  | 85+ years | 2019 | 354.51 | 468.83 | 259.81 |
| Saudi Arabia | High-income | <5 years | 2019 | 2.24 | 3.67 | 1.12 |
|  |  | 5-9 years | 2019 | 6.06 | 9.60 | 3.47 |
|  |  | 10-14 years | 2019 | 9.02 | 14.69 | 5.34 |
|  |  | 15-19 years | 2019 | 11.52 | 17.41 | 7.32 |
|  |  | 20-24 years | 2019 | 13.73 | 19.90 | 9.03 |
|  |  | 25-29 years | 2019 | 16.28 | 22.93 | 11.02 |
|  |  | 30-34 years | 2019 | 19.47 | 27.62 | 13.04 |
|  |  | 35-39 years | 2019 | 24.62 | 33.59 | 17.03 |
|  |  | 40-44 years | 2019 | 31.94 | 42.98 | 22.51 |
|  |  | 45-49 years | 2019 | 41.46 | 54.90 | 30.26 |
|  |  | 50-54 years | 2019 | 53.76 | 73.55 | 39.01 |
|  |  | 55-59 years | 2019 | 71.08 | 95.56 | 51.91 |
|  |  | 60-64 years | 2019 | 93.49 | 126.58 | 64.90 |
|  |  | 65-69 years | 2019 | 117.88 | 155.58 | 85.81 |
|  |  | 70-74 years | 2019 | 143.98 | 188.07 | 104.34 |
|  |  | 75-79 years | 2019 | 173.87 | 223.86 | 126.82 |
|  |  | 80-84 years | 2019 | 209.76 | 274.44 | 150.94 |
|  |  | 85+ years | 2019 | 268.51 | 358.30 | 196.46 |
| Somalia | Low-income | <5 years | 2019 | 68.38 | 112.43 | 35.82 |
|  |  | 5-9 years | 2019 | 449.17 | 669.97 | 277.50 |
|  |  | 10-14 years | 2019 | 1118.11 | 1805.08 | 652.07 |
|  |  | 15-19 years | 2019 | 1856.18 | 2847.87 | 1159.11 |
|  |  | 20-24 years | 2019 | 2088.87 | 2925.86 | 1421.84 |
|  |  | 25-29 years | 2019 | 2095.19 | 2831.73 | 1443.31 |
|  |  | 30-34 years | 2019 | 2002.84 | 2714.84 | 1439.69 |
|  |  | 35-39 years | 2019 | 1837.84 | 2409.24 | 1376.01 |
|  |  | 40-44 years | 2019 | 1616.24 | 2085.04 | 1241.73 |
|  |  | 45-49 years | 2019 | 1350.22 | 1711.79 | 1049.68 |
|  |  | 50-54 years | 2019 | 1057.47 | 1345.13 | 816.19 |
|  |  | 55-59 years | 2019 | 817.93 | 1059.12 | 622.52 |
|  |  | 60-64 years | 2019 | 627.49 | 821.10 | 474.21 |
|  |  | 65-69 years | 2019 | 481.59 | 633.07 | 363.13 |
|  |  | 70-74 years | 2019 | 368.64 | 486.58 | 272.82 |
|  |  | 75-79 years | 2019 | 283.60 | 379.99 | 209.83 |
|  |  | 80-84 years | 2019 | 217.19 | 294.61 | 161.01 |
|  |  | 85+ years | 2019 | 154.92 | 214.43 | 112.49 |
| Sudan | Low-income | <5 years | 2019 | 38.53 | 64.79 | 19.83 |
|  |  | 5-9 years | 2019 | 220.27 | 334.65 | 133.58 |
|  |  | 10-14 years | 2019 | 431.07 | 683.03 | 249.95 |
|  |  | 15-19 years | 2019 | 626.13 | 964.92 | 377.25 |
|  |  | 20-24 years | 2019 | 774.90 | 1062.18 | 525.32 |
|  |  | 25-29 years | 2019 | 863.36 | 1172.88 | 592.58 |
|  |  | 30-34 years | 2019 | 901.15 | 1216.39 | 643.51 |
|  |  | 35-39 years | 2019 | 898.31 | 1178.43 | 681.22 |
|  |  | 40-44 years | 2019 | 853.27 | 1090.37 | 657.85 |
|  |  | 45-49 years | 2019 | 750.90 | 956.21 | 578.91 |
|  |  | 50-54 years | 2019 | 611.97 | 781.60 | 472.31 |
|  |  | 55-59 years | 2019 | 486.72 | 633.95 | 371.08 |
|  |  | 60-64 years | 2019 | 385.77 | 505.90 | 289.49 |
|  |  | 65-69 years | 2019 | 303.35 | 396.72 | 225.99 |
|  |  | 70-74 years | 2019 | 237.76 | 313.25 | 176.36 |
|  |  | 75-79 years | 2019 | 186.61 | 249.69 | 139.81 |
|  |  | 80-84 years | 2019 | 146.98 | 202.23 | 108.34 |
|  |  | 85+ years | 2019 | 105.52 | 147.21 | 74.86 |
| Syrian Arab Republic | Middle-income | <5 years | 2019 | 36.54 | 59.16 | 18.50 |
|  |  | 5-9 years | 2019 | 208.52 | 312.67 | 127.07 |
|  |  | 10-14 years | 2019 | 403.05 | 650.99 | 237.75 |
|  |  | 15-19 years | 2019 | 576.94 | 857.22 | 356.16 |
|  |  | 20-24 years | 2019 | 712.08 | 970.53 | 495.01 |
|  |  | 25-29 years | 2019 | 798.73 | 1082.31 | 552.59 |
|  |  | 30-34 years | 2019 | 842.69 | 1129.12 | 609.51 |
|  |  | 35-39 years | 2019 | 849.95 | 1104.24 | 644.12 |
|  |  | 40-44 years | 2019 | 821.65 | 1045.21 | 639.37 |
|  |  | 45-49 years | 2019 | 736.37 | 931.88 | 570.55 |
|  |  | 50-54 years | 2019 | 608.77 | 770.69 | 474.05 |
|  |  | 55-59 years | 2019 | 489.73 | 633.43 | 375.71 |
|  |  | 60-64 years | 2019 | 389.98 | 511.05 | 299.17 |
|  |  | 65-69 years | 2019 | 308.39 | 402.91 | 232.79 |
|  |  | 70-74 years | 2019 | 242.16 | 319.73 | 183.51 |
|  |  | 75-79 years | 2019 | 189.26 | 250.47 | 141.88 |
|  |  | 80-84 years | 2019 | 146.62 | 198.99 | 109.40 |
|  |  | 85+ years | 2019 | 106.51 | 147.87 | 77.14 |
| Tunisia | Middle-income | <5 years | 2019 | 2.25 | 3.62 | 1.12 |
|  |  | 5-9 years | 2019 | 5.88 | 9.16 | 3.41 |
|  |  | 10-14 years | 2019 | 8.40 | 13.23 | 5.02 |
|  |  | 15-19 years | 2019 | 10.43 | 15.81 | 6.56 |
|  |  | 20-24 years | 2019 | 12.19 | 17.39 | 8.01 |
|  |  | 25-29 years | 2019 | 13.85 | 20.15 | 9.24 |
|  |  | 30-34 years | 2019 | 15.72 | 22.84 | 10.25 |
|  |  | 35-39 years | 2019 | 19.26 | 26.67 | 12.74 |
|  |  | 40-44 years | 2019 | 24.61 | 34.24 | 16.75 |
|  |  | 45-49 years | 2019 | 31.83 | 42.86 | 22.78 |
|  |  | 50-54 years | 2019 | 41.34 | 57.54 | 29.31 |
|  |  | 55-59 years | 2019 | 54.99 | 74.19 | 39.51 |
|  |  | 60-64 years | 2019 | 72.56 | 99.22 | 48.72 |
|  |  | 65-69 years | 2019 | 93.15 | 124.05 | 66.09 |
|  |  | 70-74 years | 2019 | 116.31 | 152.49 | 81.86 |
|  |  | 75-79 years | 2019 | 141.93 | 183.96 | 102.00 |
|  |  | 80-84 years | 2019 | 168.18 | 226.22 | 116.10 |
|  |  | 85+ years | 2019 | 199.50 | 271.99 | 145.52 |
| United Arab Emirates | High-income | <5 years | 2019 | 39.05 | 63.09 | 19.79 |
|  |  | 5-9 years | 2019 | 216.47 | 331.77 | 129.83 |
|  |  | 10-14 years | 2019 | 409.81 | 644.55 | 244.22 |
|  |  | 15-19 years | 2019 | 570.73 | 850.91 | 356.67 |
|  |  | 20-24 years | 2019 | 689.74 | 946.16 | 486.13 |
|  |  | 25-29 years | 2019 | 757.35 | 998.92 | 530.25 |
|  |  | 30-34 years | 2019 | 786.37 | 1036.23 | 579.40 |
|  |  | 35-39 years | 2019 | 817.83 | 1063.36 | 627.09 |
|  |  | 40-44 years | 2019 | 795.35 | 1015.98 | 620.10 |
|  |  | 45-49 years | 2019 | 723.26 | 915.49 | 564.71 |
|  |  | 50-54 years | 2019 | 606.29 | 773.51 | 469.23 |
|  |  | 55-59 years | 2019 | 496.96 | 635.80 | 379.93 |
|  |  | 60-64 years | 2019 | 401.38 | 525.49 | 305.13 |
|  |  | 65-69 years | 2019 | 323.37 | 428.39 | 243.85 |
|  |  | 70-74 years | 2019 | 267.01 | 355.40 | 201.34 |
|  |  | 75-79 years | 2019 | 221.20 | 300.74 | 162.27 |
|  |  | 80-84 years | 2019 | 180.44 | 249.21 | 126.12 |
|  |  | 85+ years | 2019 | 139.57 | 197.44 | 96.00 |
| Yemen | Low-income | <5 years | 2019 | 44.24 | 73.99 | 22.77 |
|  |  | 5-9 years | 2019 | 253.03 | 386.50 | 156.46 |
|  |  | 10-14 years | 2019 | 499.21 | 776.07 | 286.84 |
|  |  | 15-19 years | 2019 | 729.30 | 1150.12 | 442.48 |
|  |  | 20-24 years | 2019 | 913.35 | 1270.87 | 613.60 |
|  |  | 25-29 years | 2019 | 1019.49 | 1392.99 | 692.32 |
|  |  | 30-34 years | 2019 | 1058.41 | 1422.48 | 751.39 |
|  |  | 35-39 years | 2019 | 1045.77 | 1366.64 | 792.23 |
|  |  | 40-44 years | 2019 | 986.64 | 1263.08 | 764.68 |
|  |  | 45-49 years | 2019 | 866.58 | 1104.12 | 672.24 |
|  |  | 50-54 years | 2019 | 705.63 | 893.65 | 539.59 |
|  |  | 55-59 years | 2019 | 561.92 | 725.87 | 430.63 |
|  |  | 60-64 years | 2019 | 444.68 | 582.16 | 335.01 |
|  |  | 65-69 years | 2019 | 349.46 | 460.97 | 261.27 |
|  |  | 70-74 years | 2019 | 273.64 | 364.41 | 204.43 |
|  |  | 75-79 years | 2019 | 214.19 | 292.46 | 159.19 |
|  |  | 80-84 years | 2019 | 167.23 | 228.87 | 122.56 |
|  |  | 85+ years | 2019 | 121.41 | 170.29 | 87.10 |

**Supplementary Table 1C. Incidence rate estimates with uncertainty intervals from IHME GBD 2019**

| Country | Group | Age | year | Incidence (Per 100,000) | Upper value | Lower value |
| --- | --- | --- | --- | --- | --- | --- |
| Afghanistan | Low-income | <5 years | 2019 | 22.88 | 37.74 | 11.59 |
|  |  | 5-9 years | 2019 | 52.76 | 86.66 | 28.27 |
|  |  | 10-14 years | 2019 | 61.96 | 113.32 | 28.98 |
|  |  | 15-19 years | 2019 | 69.10 | 109.16 | 36.30 |
|  |  | 20-24 years | 2019 | 67.00 | 109.49 | 30.49 |
|  |  | 25-29 years | 2019 | 58.17 | 82.72 | 33.39 |
|  |  | 30-34 years | 2019 | 49.41 | 65.07 | 33.16 |
|  |  | 35-39 years | 2019 | 40.69 | 61.34 | 25.35 |
|  |  | 40-44 years | 2019 | 27.22 | 46.63 | 11.37 |
|  |  | 45-49 years | 2019 | 9.09 | 15.56 | 3.79 |
|  |  | 50-54 years | 2019 | 0.00 | 0.00 | 0.00 |
|  |  | 55-59 years | 2019 | 0.00 | 0.00 | 0.00 |
|  |  | 60-64 years | 2019 | 0.00 | 0.00 | 0.00 |
|  |  | 65-69 years | 2019 | 0.00 | 0.00 | 0.00 |
|  |  | 70-74 years | 2019 | 0.00 | 0.00 | 0.00 |
|  |  | 75-79 years | 2019 | 0.00 | 0.00 | 0.00 |
|  |  | 80-84 years | 2019 | 0.00 | 0.00 | 0.00 |
|  |  | 85+ years | 2019 | 0.00 | 0.00 | 0.00 |
| Bahrain | High-income | <5 years | 2019 | 0.70 | 1.12 | 0.36 |
|  |  | 5-9 years | 2019 | 0.68 | 1.31 | 0.25 |
|  |  | 10-14 years | 2019 | 0.60 | 1.25 | 0.17 |
|  |  | 15-19 years | 2019 | 0.56 | 1.11 | 0.15 |
|  |  | 20-24 years | 2019 | 0.59 | 1.36 | 0.19 |
|  |  | 25-29 years | 2019 | 0.69 | 1.49 | 0.20 |
|  |  | 30-34 years | 2019 | 0.96 | 1.71 | 0.42 |
|  |  | 35-39 years | 2019 | 1.38 | 2.45 | 0.57 |
|  |  | 40-44 years | 2019 | 1.98 | 3.12 | 1.06 |
|  |  | 45-49 years | 2019 | 2.74 | 4.83 | 1.32 |
|  |  | 50-54 years | 2019 | 4.08 | 6.46 | 2.39 |
|  |  | 55-59 years | 2019 | 6.05 | 10.04 | 2.95 |
|  |  | 60-64 years | 2019 | 7.98 | 11.53 | 4.90 |
|  |  | 65-69 years | 2019 | 9.95 | 15.61 | 5.10 |
|  |  | 70-74 years | 2019 | 13.76 | 20.29 | 8.08 |
|  |  | 75-79 years | 2019 | 19.48 | 30.83 | 11.09 |
|  |  | 80-84 years | 2019 | 25.55 | 36.20 | 17.55 |
|  |  | 85+ years | 2019 | 32.31 | 48.18 | 20.45 |
| Djibouti | Low-income | <5 years | 2019 | 42.93 | 70.35 | 22.55 |
|  |  | 5-9 years | 2019 | 129.32 | 230.34 | 63.70 |
|  |  | 10-14 years | 2019 | 202.47 | 324.11 | 112.36 |
|  |  | 15-19 years | 2019 | 176.08 | 268.26 | 85.90 |
|  |  | 20-24 years | 2019 | 107.15 | 170.98 | 52.54 |
|  |  | 25-29 years | 2019 | 89.12 | 134.07 | 49.69 |
|  |  | 30-34 years | 2019 | 70.95 | 98.37 | 45.57 |
|  |  | 35-39 years | 2019 | 52.53 | 78.17 | 33.63 |
|  |  | 40-44 years | 2019 | 32.54 | 56.75 | 15.71 |
|  |  | 45-49 years | 2019 | 10.88 | 18.96 | 5.26 |
|  |  | 50-54 years | 2019 | 0.00 | 0.00 | 0.00 |
|  |  | 55-59 years | 2019 | 0.00 | 0.00 | 0.00 |
|  |  | 60-64 years | 2019 | 0.00 | 0.00 | 0.00 |
|  |  | 65-69 years | 2019 | 0.00 | 0.00 | 0.00 |
|  |  | 70-74 years | 2019 | 0.00 | 0.00 | 0.00 |
|  |  | 75-79 years | 2019 | 0.00 | 0.00 | 0.00 |
|  |  | 80-84 years | 2019 | 0.00 | 0.00 | 0.00 |
|  |  | 85+ years | 2019 | 0.00 | 0.00 | 0.00 |
| Egypt | Middle-income | <5 years | 2019 | 23.86 | 38.75 | 12.35 |
|  |  | 5-9 years | 2019 | 54.11 | 90.65 | 29.00 |
|  |  | 10-14 years | 2019 | 62.94 | 109.58 | 30.26 |
|  |  | 15-19 years | 2019 | 64.30 | 99.41 | 32.91 |
|  |  | 20-24 years | 2019 | 57.22 | 89.01 | 28.09 |
|  |  | 25-29 years | 2019 | 48.33 | 69.74 | 28.81 |
|  |  | 30-34 years | 2019 | 39.49 | 53.10 | 25.78 |
|  |  | 35-39 years | 2019 | 30.69 | 45.43 | 18.78 |
|  |  | 40-44 years | 2019 | 19.72 | 34.41 | 8.55 |
|  |  | 45-49 years | 2019 | 6.58 | 11.48 | 2.86 |
|  |  | 50-54 years | 2019 | 0.00 | 0.00 | 0.00 |
|  |  | 55-59 years | 2019 | 0.00 | 0.00 | 0.00 |
|  |  | 60-64 years | 2019 | 0.00 | 0.00 | 0.00 |
|  |  | 65-69 years | 2019 | 0.00 | 0.00 | 0.00 |
|  |  | 70-74 years | 2019 | 0.00 | 0.00 | 0.00 |
|  |  | 75-79 years | 2019 | 0.00 | 0.00 | 0.00 |
|  |  | 80-84 years | 2019 | 0.00 | 0.00 | 0.00 |
|  |  | 85+ years | 2019 | 0.00 | 0.00 | 0.00 |
| Iran, Islamic Republic of | Middle-income | <5 years | 2019 | 19.14 | 31.72 | 9.79 |
|  |  | 5-9 years | 2019 | 48.70 | 81.76 | 25.03 |
|  |  | 10-14 years | 2019 | 61.86 | 102.39 | 32.15 |
|  |  | 15-19 years | 2019 | 48.68 | 76.32 | 25.27 |
|  |  | 20-24 years | 2019 | 59.00 | 90.97 | 30.31 |
|  |  | 25-29 years | 2019 | 41.24 | 59.62 | 25.17 |
|  |  | 30-34 years | 2019 | 33.47 | 45.14 | 22.53 |
|  |  | 35-39 years | 2019 | 25.77 | 37.85 | 15.94 |
|  |  | 40-44 years | 2019 | 16.56 | 28.16 | 7.40 |
|  |  | 45-49 years | 2019 | 5.61 | 9.47 | 2.56 |
|  |  | 50-54 years | 2019 | 0.14 | 0.20 | 0.08 |
|  |  | 55-59 years | 2019 | 0.19 | 0.30 | 0.10 |
|  |  | 60-64 years | 2019 | 0.24 | 0.34 | 0.15 |
|  |  | 65-69 years | 2019 | 0.31 | 0.46 | 0.16 |
|  |  | 70-74 years | 2019 | 0.41 | 0.60 | 0.24 |
|  |  | 75-79 years | 2019 | 0.54 | 0.86 | 0.31 |
|  |  | 80-84 years | 2019 | 0.67 | 0.94 | 0.46 |
|  |  | 85+ years | 2019 | 0.89 | 1.28 | 0.57 |
| Iraq | Middle-income | <5 years | 2019 | 23.06 | 38.33 | 11.89 |
|  |  | 5-9 years | 2019 | 52.63 | 87.10 | 28.36 |
|  |  | 10-14 years | 2019 | 60.18 | 103.83 | 29.54 |
|  |  | 15-19 years | 2019 | 60.99 | 95.85 | 31.73 |
|  |  | 20-24 years | 2019 | 54.72 | 86.53 | 27.32 |
|  |  | 25-29 years | 2019 | 46.66 | 67.74 | 27.28 |
|  |  | 30-34 years | 2019 | 38.62 | 51.97 | 25.24 |
|  |  | 35-39 years | 2019 | 30.61 | 45.91 | 18.65 |
|  |  | 40-44 years | 2019 | 19.96 | 34.05 | 8.38 |
|  |  | 45-49 years | 2019 | 6.66 | 11.36 | 2.80 |
|  |  | 50-54 years | 2019 | 0.00 | 0.00 | 0.00 |
|  |  | 55-59 years | 2019 | 0.00 | 0.00 | 0.00 |
|  |  | 60-64 years | 2019 | 0.00 | 0.00 | 0.00 |
|  |  | 65-69 years | 2019 | 0.00 | 0.00 | 0.00 |
|  |  | 70-74 years | 2019 | 0.00 | 0.00 | 0.00 |
|  |  | 75-79 years | 2019 | 0.00 | 0.00 | 0.00 |
|  |  | 80-84 years | 2019 | 0.00 | 0.00 | 0.00 |
|  |  | 85+ years | 2019 | 0.00 | 0.00 | 0.00 |
| Jordan | Middle-income | <5 years | 2019 | 0.63 | 1.00 | 0.31 |
|  |  | 5-9 years | 2019 | 0.61 | 1.14 | 0.24 |
|  |  | 10-14 years | 2019 | 0.53 | 1.10 | 0.16 |
|  |  | 15-19 years | 2019 | 0.50 | 1.03 | 0.15 |
|  |  | 20-24 years | 2019 | 0.55 | 1.20 | 0.15 |
|  |  | 25-29 years | 2019 | 0.64 | 1.37 | 0.17 |
|  |  | 30-34 years | 2019 | 0.90 | 1.66 | 0.38 |
|  |  | 35-39 years | 2019 | 1.36 | 2.49 | 0.58 |
|  |  | 40-44 years | 2019 | 1.84 | 2.94 | 0.94 |
|  |  | 45-49 years | 2019 | 2.35 | 4.07 | 1.09 |
|  |  | 50-54 years | 2019 | 2.87 | 4.49 | 1.54 |
|  |  | 55-59 years | 2019 | 3.39 | 5.60 | 1.67 |
|  |  | 60-64 years | 2019 | 3.89 | 5.99 | 2.01 |
|  |  | 65-69 years | 2019 | 4.35 | 6.75 | 2.21 |
|  |  | 70-74 years | 2019 | 5.27 | 8.05 | 2.97 |
|  |  | 75-79 years | 2019 | 6.61 | 10.57 | 3.52 |
|  |  | 80-84 years | 2019 | 8.59 | 12.28 | 5.36 |
|  |  | 85+ years | 2019 | 12.63 | 18.69 | 8.11 |
| Kuwait | High-income | <5 years | 2019 | 0.64 | 1.02 | 0.33 |
|  |  | 5-9 years | 2019 | 0.62 | 1.16 | 0.26 |
|  |  | 10-14 years | 2019 | 0.53 | 1.13 | 0.17 |
|  |  | 15-19 years | 2019 | 0.49 | 1.02 | 0.14 |
|  |  | 20-24 years | 2019 | 0.53 | 1.20 | 0.14 |
|  |  | 25-29 years | 2019 | 0.63 | 1.36 | 0.17 |
|  |  | 30-34 years | 2019 | 0.90 | 1.64 | 0.38 |
|  |  | 35-39 years | 2019 | 1.31 | 2.37 | 0.48 |
|  |  | 40-44 years | 2019 | 1.81 | 2.99 | 0.86 |
|  |  | 45-49 years | 2019 | 2.40 | 4.29 | 1.09 |
|  |  | 50-54 years | 2019 | 3.19 | 5.13 | 1.70 |
|  |  | 55-59 years | 2019 | 4.20 | 7.19 | 1.88 |
|  |  | 60-64 years | 2019 | 5.10 | 7.96 | 2.53 |
|  |  | 65-69 years | 2019 | 5.93 | 9.27 | 2.74 |
|  |  | 70-74 years | 2019 | 7.38 | 11.02 | 4.03 |
|  |  | 75-79 years | 2019 | 9.80 | 15.65 | 5.12 |
|  |  | 80-84 years | 2019 | 12.42 | 18.20 | 7.84 |
|  |  | 85+ years | 2019 | 19.02 | 28.59 | 11.98 |
| Lebanon | Middle-income | <5 years | 2019 | 0.73 | 1.16 | 0.39 |
|  |  | 5-9 years | 2019 | 0.77 | 1.46 | 0.30 |
|  |  | 10-14 years | 2019 | 0.71 | 1.38 | 0.23 |
|  |  | 15-19 years | 2019 | 0.68 | 1.31 | 0.23 |
|  |  | 20-24 years | 2019 | 0.72 | 1.54 | 0.24 |
|  |  | 25-29 years | 2019 | 0.84 | 1.69 | 0.24 |
|  |  | 30-34 years | 2019 | 1.24 | 2.13 | 0.57 |
|  |  | 35-39 years | 2019 | 1.91 | 3.02 | 0.99 |
|  |  | 40-44 years | 2019 | 2.52 | 3.78 | 1.50 |
|  |  | 45-49 years | 2019 | 3.10 | 5.37 | 1.55 |
|  |  | 50-54 years | 2019 | 4.09 | 6.33 | 2.33 |
|  |  | 55-59 years | 2019 | 5.51 | 8.98 | 2.70 |
|  |  | 60-64 years | 2019 | 6.96 | 10.30 | 4.02 |
|  |  | 65-69 years | 2019 | 8.50 | 12.83 | 3.84 |
|  |  | 70-74 years | 2019 | 10.54 | 15.87 | 5.81 |
|  |  | 75-79 years | 2019 | 13.05 | 20.59 | 6.92 |
|  |  | 80-84 years | 2019 | 16.40 | 23.77 | 10.39 |
|  |  | 85+ years | 2019 | 22.55 | 33.68 | 14.28 |
| Libya | Middle-income | <5 years | 2019 | 23.39 | 39.08 | 11.89 |
|  |  | 5-9 years | 2019 | 51.80 | 86.51 | 28.33 |
|  |  | 10-14 years | 2019 | 57.42 | 98.78 | 27.62 |
|  |  | 15-19 years | 2019 | 56.54 | 87.61 | 29.14 |
|  |  | 20-24 years | 2019 | 49.93 | 79.69 | 25.21 |
|  |  | 25-29 years | 2019 | 42.49 | 63.16 | 25.03 |
|  |  | 30-34 years | 2019 | 35.09 | 47.80 | 23.19 |
|  |  | 35-39 years | 2019 | 27.71 | 41.53 | 16.93 |
|  |  | 40-44 years | 2019 | 18.02 | 31.30 | 7.90 |
|  |  | 45-49 years | 2019 | 6.01 | 10.43 | 2.63 |
|  |  | 50-54 years | 2019 | 0.00 | 0.00 | 0.00 |
|  |  | 55-59 years | 2019 | 0.00 | 0.00 | 0.00 |
|  |  | 60-64 years | 2019 | 0.00 | 0.00 | 0.00 |
|  |  | 65-69 years | 2019 | 0.00 | 0.00 | 0.00 |
|  |  | 70-74 years | 2019 | 0.00 | 0.00 | 0.00 |
|  |  | 75-79 years | 2019 | 0.00 | 0.00 | 0.00 |
|  |  | 80-84 years | 2019 | 0.00 | 0.00 | 0.00 |
|  |  | 85+ years | 2019 | 0.00 | 0.00 | 0.00 |
| Morocco | Middle-income | <5 years | 2019 | 23.55 | 39.21 | 12.20 |
|  |  | 5-9 years | 2019 | 53.21 | 88.59 | 29.03 |
|  |  | 10-14 years | 2019 | 60.88 | 106.85 | 29.46 |
|  |  | 15-19 years | 2019 | 62.63 | 97.31 | 31.67 |
|  |  | 20-24 years | 2019 | 56.83 | 90.67 | 27.16 |
|  |  | 25-29 years | 2019 | 48.17 | 72.24 | 28.13 |
|  |  | 30-34 years | 2019 | 39.52 | 53.98 | 26.30 |
|  |  | 35-39 years | 2019 | 30.90 | 46.72 | 19.03 |
|  |  | 40-44 years | 2019 | 19.94 | 35.07 | 8.57 |
|  |  | 45-49 years | 2019 | 6.65 | 11.70 | 2.86 |
|  |  | 50-54 years | 2019 | 0.00 | 0.00 | 0.00 |
|  |  | 55-59 years | 2019 | 0.00 | 0.00 | 0.00 |
|  |  | 60-64 years | 2019 | 0.00 | 0.00 | 0.00 |
|  |  | 65-69 years | 2019 | 0.00 | 0.00 | 0.00 |
|  |  | 70-74 years | 2019 | 0.00 | 0.00 | 0.00 |
|  |  | 75-79 years | 2019 | 0.00 | 0.00 | 0.00 |
|  |  | 80-84 years | 2019 | 0.00 | 0.00 | 0.00 |
|  |  | 85+ years | 2019 | 0.00 | 0.00 | 0.00 |
| Oman | High-income | <5 years | 2019 | 0.70 | 1.12 | 0.38 |
|  |  | 5-9 years | 2019 | 0.68 | 1.35 | 0.27 |
|  |  | 10-14 years | 2019 | 0.58 | 1.21 | 0.19 |
|  |  | 15-19 years | 2019 | 0.56 | 1.11 | 0.17 |
|  |  | 20-24 years | 2019 | 0.59 | 1.29 | 0.18 |
|  |  | 25-29 years | 2019 | 0.67 | 1.45 | 0.19 |
|  |  | 30-34 years | 2019 | 0.95 | 1.70 | 0.42 |
|  |  | 35-39 years | 2019 | 1.42 | 2.55 | 0.53 |
|  |  | 40-44 years | 2019 | 2.00 | 3.31 | 0.96 |
|  |  | 45-49 years | 2019 | 2.66 | 4.77 | 1.24 |
|  |  | 50-54 years | 2019 | 3.66 | 5.74 | 2.07 |
|  |  | 55-59 years | 2019 | 5.04 | 8.56 | 2.37 |
|  |  | 60-64 years | 2019 | 6.80 | 10.18 | 3.84 |
|  |  | 65-69 years | 2019 | 9.14 | 14.16 | 4.72 |
|  |  | 70-74 years | 2019 | 11.34 | 16.45 | 6.67 |
|  |  | 75-79 years | 2019 | 13.93 | 22.25 | 7.76 |
|  |  | 80-84 years | 2019 | 17.14 | 25.12 | 10.75 |
|  |  | 85+ years | 2019 | 23.35 | 35.05 | 14.33 |
| Pakistan | Low-income | <5 years | 2019 | 18.87 | 31.30 | 9.82 |
|  |  | 5-9 years | 2019 | 54.41 | 94.90 | 26.38 |
|  |  | 10-14 years | 2019 | 89.04 | 152.77 | 46.73 |
|  |  | 15-19 years | 2019 | 121.57 | 174.16 | 73.05 |
|  |  | 20-24 years | 2019 | 121.18 | 178.83 | 69.32 |
|  |  | 25-29 years | 2019 | 95.07 | 134.08 | 59.80 |
|  |  | 30-34 years | 2019 | 69.03 | 91.17 | 46.94 |
|  |  | 35-39 years | 2019 | 43.06 | 61.10 | 28.78 |
|  |  | 40-44 years | 2019 | 22.54 | 40.42 | 9.92 |
|  |  | 45-49 years | 2019 | 7.53 | 13.48 | 3.32 |
|  |  | 50-54 years | 2019 | 0.00 | 0.00 | 0.00 |
|  |  | 55-59 years | 2019 | 0.00 | 0.00 | 0.00 |
|  |  | 60-64 years | 2019 | 0.00 | 0.00 | 0.00 |
|  |  | 65-69 years | 2019 | 0.00 | 0.00 | 0.00 |
|  |  | 70-74 years | 2019 | 0.00 | 0.00 | 0.00 |
|  |  | 75-79 years | 2019 | 0.00 | 0.00 | 0.00 |
|  |  | 80-84 years | 2019 | 0.00 | 0.00 | 0.00 |
|  |  | 85+ years | 2019 | 0.00 | 0.00 | 0.00 |
| Occupied Palestinian territory | Middle-income | <5 years | 2019 | 22.19 | 36.47 | 11.18 |
|  |  | 5-9 years | 2019 | 50.37 | 84.43 | 27.64 |
|  |  | 10-14 years | 2019 | 56.23 | 98.76 | 27.25 |
|  |  | 15-19 years | 2019 | 55.05 | 85.48 | 28.40 |
|  |  | 20-24 years | 2019 | 48.71 | 77.97 | 23.96 |
|  |  | 25-29 years | 2019 | 41.52 | 62.10 | 24.09 |
|  |  | 30-34 years | 2019 | 34.34 | 46.71 | 22.58 |
|  |  | 35-39 years | 2019 | 27.19 | 40.84 | 16.89 |
|  |  | 40-44 years | 2019 | 17.72 | 30.53 | 8.09 |
|  |  | 45-49 years | 2019 | 5.91 | 10.21 | 2.70 |
|  |  | 50-54 years | 2019 | 0.00 | 0.00 | 0.00 |
|  |  | 55-59 years | 2019 | 0.00 | 0.00 | 0.00 |
|  |  | 60-64 years | 2019 | 0.00 | 0.00 | 0.00 |
|  |  | 65-69 years | 2019 | 0.00 | 0.00 | 0.00 |
|  |  | 70-74 years | 2019 | 0.00 | 0.00 | 0.00 |
|  |  | 75-79 years | 2019 | 0.00 | 0.00 | 0.00 |
|  |  | 80-84 years | 2019 | 0.00 | 0.00 | 0.00 |
|  |  | 85+ years | 2019 | 0.00 | 0.00 | 0.00 |
| Qatar | High-income | <5 years | 2019 | 0.65 | 1.04 | 0.33 |
|  |  | 5-9 years | 2019 | 0.61 | 1.17 | 0.25 |
|  |  | 10-14 years | 2019 | 0.53 | 1.10 | 0.16 |
|  |  | 15-19 years | 2019 | 0.48 | 1.00 | 0.13 |
|  |  | 20-24 years | 2019 | 0.46 | 1.05 | 0.11 |
|  |  | 25-29 years | 2019 | 0.55 | 1.21 | 0.14 |
|  |  | 30-34 years | 2019 | 0.85 | 1.54 | 0.36 |
|  |  | 35-39 years | 2019 | 1.35 | 2.43 | 0.56 |
|  |  | 40-44 years | 2019 | 1.99 | 3.10 | 1.00 |
|  |  | 45-49 years | 2019 | 2.78 | 4.85 | 1.23 |
|  |  | 50-54 years | 2019 | 3.99 | 6.39 | 2.21 |
|  |  | 55-59 years | 2019 | 5.59 | 9.90 | 2.53 |
|  |  | 60-64 years | 2019 | 7.63 | 11.72 | 4.14 |
|  |  | 65-69 years | 2019 | 10.28 | 16.92 | 4.62 |
|  |  | 70-74 years | 2019 | 14.81 | 22.05 | 8.77 |
|  |  | 75-79 years | 2019 | 22.50 | 33.75 | 12.85 |
|  |  | 80-84 years | 2019 | 24.07 | 34.43 | 15.42 |
|  |  | 85+ years | 2019 | 22.81 | 35.21 | 13.46 |
| Saudi Arabia | High-income | <5 years | 2019 | 0.70 | 1.13 | 0.38 |
|  |  | 5-9 years | 2019 | 0.72 | 1.36 | 0.27 |
|  |  | 10-14 years | 2019 | 0.68 | 1.37 | 0.23 |
|  |  | 15-19 years | 2019 | 0.67 | 1.27 | 0.24 |
|  |  | 20-24 years | 2019 | 0.79 | 1.58 | 0.30 |
|  |  | 25-29 years | 2019 | 1.03 | 1.94 | 0.41 |
|  |  | 30-34 years | 2019 | 1.52 | 2.38 | 0.89 |
|  |  | 35-39 years | 2019 | 2.25 | 3.56 | 1.26 |
|  |  | 40-44 years | 2019 | 2.88 | 4.27 | 1.81 |
|  |  | 45-49 years | 2019 | 3.44 | 5.88 | 1.75 |
|  |  | 50-54 years | 2019 | 4.53 | 6.81 | 2.56 |
|  |  | 55-59 years | 2019 | 6.12 | 10.00 | 3.10 |
|  |  | 60-64 years | 2019 | 7.46 | 11.09 | 4.52 |
|  |  | 65-69 years | 2019 | 8.52 | 12.51 | 4.36 |
|  |  | 70-74 years | 2019 | 9.87 | 14.52 | 5.38 |
|  |  | 75-79 years | 2019 | 11.61 | 18.11 | 6.33 |
|  |  | 80-84 years | 2019 | 14.10 | 20.67 | 8.90 |
|  |  | 85+ years | 2019 | 19.42 | 29.64 | 11.94 |
| Somalia | Low-income | <5 years | 2019 | 42.40 | 69.39 | 22.26 |
|  |  | 5-9 years | 2019 | 133.80 | 240.57 | 64.51 |
|  |  | 10-14 years | 2019 | 224.84 | 371.90 | 124.81 |
|  |  | 15-19 years | 2019 | 210.51 | 327.23 | 102.29 |
|  |  | 20-24 years | 2019 | 137.72 | 229.42 | 65.45 |
|  |  | 25-29 years | 2019 | 114.28 | 174.21 | 63.35 |
|  |  | 30-34 years | 2019 | 90.85 | 120.96 | 57.45 |
|  |  | 35-39 years | 2019 | 67.43 | 99.33 | 41.60 |
|  |  | 40-44 years | 2019 | 41.83 | 74.09 | 18.54 |
|  |  | 45-49 years | 2019 | 13.97 | 24.73 | 6.20 |
|  |  | 50-54 years | 2019 | 0.00 | 0.00 | 0.00 |
|  |  | 55-59 years | 2019 | 0.00 | 0.00 | 0.00 |
|  |  | 60-64 years | 2019 | 0.00 | 0.00 | 0.00 |
|  |  | 65-69 years | 2019 | 0.00 | 0.00 | 0.00 |
|  |  | 70-74 years | 2019 | 0.00 | 0.00 | 0.00 |
|  |  | 75-79 years | 2019 | 0.00 | 0.00 | 0.00 |
|  |  | 80-84 years | 2019 | 0.00 | 0.00 | 0.00 |
|  |  | 85+ years | 2019 | 0.00 | 0.00 | 0.00 |
| Sudan | Low-income | <5 years | 2019 | 23.82 | 39.95 | 12.35 |
|  |  | 5-9 years | 2019 | 55.65 | 94.01 | 30.07 |
|  |  | 10-14 years | 2019 | 65.21 | 114.67 | 30.68 |
|  |  | 15-19 years | 2019 | 69.29 | 106.76 | 36.12 |
|  |  | 20-24 years | 2019 | 64.54 | 103.82 | 31.46 |
|  |  | 25-29 years | 2019 | 54.82 | 80.29 | 31.86 |
|  |  | 30-34 years | 2019 | 45.05 | 60.57 | 29.44 |
|  |  | 35-39 years | 2019 | 35.18 | 53.52 | 21.38 |
|  |  | 40-44 years | 2019 | 22.70 | 40.24 | 9.39 |
|  |  | 45-49 years | 2019 | 7.59 | 13.38 | 3.13 |
|  |  | 50-54 years | 2019 | 0.00 | 0.00 | 0.00 |
|  |  | 55-59 years | 2019 | 0.00 | 0.00 | 0.00 |
|  |  | 60-64 years | 2019 | 0.00 | 0.00 | 0.00 |
|  |  | 65-69 years | 2019 | 0.00 | 0.00 | 0.00 |
|  |  | 70-74 years | 2019 | 0.00 | 0.00 | 0.00 |
|  |  | 75-79 years | 2019 | 0.00 | 0.00 | 0.00 |
|  |  | 80-84 years | 2019 | 0.00 | 0.00 | 0.00 |
|  |  | 85+ years | 2019 | 0.00 | 0.00 | 0.00 |
| Syrian Arab Republic | Middle-income | <5 years | 2019 | 22.24 | 35.96 | 11.31 |
|  |  | 5-9 years | 2019 | 50.60 | 84.95 | 27.50 |
|  |  | 10-14 years | 2019 | 57.71 | 100.54 | 28.02 |
|  |  | 15-19 years | 2019 | 58.79 | 91.49 | 30.06 |
|  |  | 20-24 years | 2019 | 53.02 | 83.78 | 27.79 |
|  |  | 25-29 years | 2019 | 44.99 | 66.17 | 27.67 |
|  |  | 30-34 years | 2019 | 36.97 | 51.22 | 24.65 |
|  |  | 35-39 years | 2019 | 29.01 | 43.79 | 17.84 |
|  |  | 40-44 years | 2019 | 18.81 | 33.02 | 8.28 |
|  |  | 45-49 years | 2019 | 6.28 | 11.02 | 2.76 |
|  |  | 50-54 years | 2019 | 0.00 | 0.00 | 0.00 |
|  |  | 55-59 years | 2019 | 0.00 | 0.00 | 0.00 |
|  |  | 60-64 years | 2019 | 0.00 | 0.00 | 0.00 |
|  |  | 65-69 years | 2019 | 0.00 | 0.00 | 0.00 |
|  |  | 70-74 years | 2019 | 0.00 | 0.00 | 0.00 |
|  |  | 75-79 years | 2019 | 0.00 | 0.00 | 0.00 |
|  |  | 80-84 years | 2019 | 0.00 | 0.00 | 0.00 |
|  |  | 85+ years | 2019 | 0.00 | 0.00 | 0.00 |
| Tunisia | Middle-income | <5 years | 2019 | 0.72 | 1.13 | 0.39 |
|  |  | 5-9 years | 2019 | 0.68 | 1.27 | 0.28 |
|  |  | 10-14 years | 2019 | 0.58 | 1.25 | 0.18 |
|  |  | 15-19 years | 2019 | 0.56 | 1.14 | 0.16 |
|  |  | 20-24 years | 2019 | 0.61 | 1.35 | 0.19 |
|  |  | 25-29 years | 2019 | 0.71 | 1.47 | 0.22 |
|  |  | 30-34 years | 2019 | 1.03 | 1.79 | 0.49 |
|  |  | 35-39 years | 2019 | 1.56 | 2.60 | 0.72 |
|  |  | 40-44 years | 2019 | 2.13 | 3.23 | 1.15 |
|  |  | 45-49 years | 2019 | 2.73 | 4.80 | 1.28 |
|  |  | 50-54 years | 2019 | 3.74 | 5.71 | 2.12 |
|  |  | 55-59 years | 2019 | 5.15 | 8.45 | 2.61 |
|  |  | 60-64 years | 2019 | 6.61 | 9.60 | 4.03 |
|  |  | 65-69 years | 2019 | 8.14 | 12.03 | 4.16 |
|  |  | 70-74 years | 2019 | 10.38 | 14.86 | 6.06 |
|  |  | 75-79 years | 2019 | 13.35 | 20.70 | 7.79 |
|  |  | 80-84 years | 2019 | 17.19 | 24.12 | 11.89 |
|  |  | 85+ years | 2019 | 23.93 | 34.59 | 16.03 |
| United Arab Emirates | High-income | <5 years | 2019 | 23.17 | 37.82 | 11.76 |
|  |  | 5-9 years | 2019 | 48.65 | 80.17 | 26.98 |
|  |  | 10-14 years | 2019 | 52.16 | 91.30 | 25.02 |
|  |  | 15-19 years | 2019 | 50.62 | 80.05 | 26.39 |
|  |  | 20-24 years | 2019 | 44.36 | 70.47 | 22.47 |
|  |  | 25-29 years | 2019 | 38.06 | 56.26 | 23.10 |
|  |  | 30-34 years | 2019 | 31.84 | 43.36 | 21.14 |
|  |  | 35-39 years | 2019 | 25.34 | 37.91 | 15.18 |
|  |  | 40-44 years | 2019 | 16.70 | 28.15 | 7.60 |
|  |  | 45-49 years | 2019 | 5.57 | 9.38 | 2.54 |
|  |  | 50-54 years | 2019 | 0.00 | 0.00 | 0.00 |
|  |  | 55-59 years | 2019 | 0.00 | 0.00 | 0.00 |
|  |  | 60-64 years | 2019 | 0.00 | 0.00 | 0.00 |
|  |  | 65-69 years | 2019 | 0.00 | 0.00 | 0.00 |
|  |  | 70-74 years | 2019 | 0.00 | 0.00 | 0.00 |
|  |  | 75-79 years | 2019 | 0.00 | 0.00 | 0.00 |
|  |  | 80-84 years | 2019 | 0.00 | 0.00 | 0.00 |
|  |  | 85+ years | 2019 | 0.00 | 0.00 | 0.00 |
| Yemen | Low-income | <5 years | 2019 | 27.36 | 45.58 | 14.00 |
|  |  | 5-9 years | 2019 | 64.35 | 106.92 | 34.47 |
|  |  | 10-14 years | 2019 | 76.46 | 137.60 | 36.50 |
|  |  | 15-19 years | 2019 | 83.57 | 130.13 | 43.01 |
|  |  | 20-24 years | 2019 | 79.38 | 127.03 | 38.63 |
|  |  | 25-29 years | 2019 | 66.68 | 98.23 | 38.18 |
|  |  | 30-34 years | 2019 | 54.02 | 73.20 | 34.86 |
|  |  | 35-39 years | 2019 | 41.40 | 62.16 | 25.26 |
|  |  | 40-44 years | 2019 | 26.35 | 46.49 | 10.59 |
|  |  | 45-49 years | 2019 | 8.79 | 15.50 | 3.53 |
|  |  | 50-54 years | 2019 | 0.00 | 0.00 | 0.00 |
|  |  | 55-59 years | 2019 | 0.00 | 0.00 | 0.00 |
|  |  | 60-64 years | 2019 | 0.00 | 0.00 | 0.00 |
|  |  | 65-69 years | 2019 | 0.00 | 0.00 | 0.00 |
|  |  | 70-74 years | 2019 | 0.00 | 0.00 | 0.00 |
|  |  | 75-79 years | 2019 | 0.00 | 0.00 | 0.00 |
|  |  | 80-84 years | 2019 | 0.00 | 0.00 | 0.00 |
|  |  | 85+ years | 2019 | 0.00 | 0.00 | 0.00 |

**Supplementary Materials 2. Assumptions and Data Sources**

**Assumptions**

- The baseline year for the study was set as 2020. Population forecasts obtained from UN World Population Prospects from 2021-2050.
- Epidemiological indicators such as prevalence and mortality are assumed to remain the same from 2021-2050, same as the base year.
- Direct costs include costs related to treating ARF, performing valve surgeries, and treating complications of RHD which include atrial fibrillation, infective endocarditis and stroke.
- The treatment coverage rates for valve surgery were obtained from the literature. The average treatment cost of ARF, valve surgery, and post-stroke were estimated using a cost-ingredient method, which includes the cost of drugs and supplies, (2) outpatient visits, and (3) inpatient visits. The drug and supply costs were extracted from the OneHealth Tool. The costs of outpatient and inpatient visits were calculated by multiplying assumptions on the average number of visits extracted from the OneHealth Tool ^1^ and average visit costs from the WHO-CHOICE database.
- The treatment costs related to atrial fibrillation, infective endocarditis, and heart failure, we estimated these costs as a proportion of post-stroke treatment costs using ratios derived from available literature.
- Productivity indicators were assumed to remain constant during the study period, due to large levels of uncertainty surrounding these variables.
- For calculating absenteeism, it was assumed that, at the very least, a patient misses work on the days that they require an outpatient or inpatient visit. In order to estimate the absenteeism-related productivity reduction coefficient, the annual working days missed were divided by the average number of working days in each country.
- For caregiver absenteeism, it was assumed that, at the very least, a caregiver misses work on the days on which their child requires an outpatient or inpatient visit.
- To calculate the loss of future productivity, this study used two assumptions regarding (a) the impact of RHD on schooling and (b) the economic consequences of reduced schooling. It was assumed that 40% of children and teenagers affected by RHD are behind by at least one grade. It was also assumed that one year of school loss translated into a decrease in future earnings between 0.70% and 8.30%, depending on the country.
- The working years in a lifetime were estimated at 54, assuming that children will work from 15 to 69.
- A discount rate of 3% was used.

**Supplementary Table 2. Data Sources**

| **Parameters** | **Data sources** |
| --- | --- |
| Total Population, population-15+, Population-15-64 years. | World Bank Database ^2^ |
| RHD Prevalence | Global Burden of Disease (2019) ^3^ |
| RHD Incidence | Global Burden of Disease (2019) ^3^ |
| *Average cost per patient for RHD* | Figueiredo et al. (2019) ^4^ |
| *Average cost of Inpatient visits* | WHO CHOICE^5^ |
| Mortality due to RHD | Global Burden of Disease (2019) ^3^ |
| Life Expectancy | WHO Global Health Observatory – Life Expectancy ^6^ |
| Absenteeism | OneHealth Tool ^1^ Kotseva et al. (2020) ^7^ |
| GDP (in USD) | World Bank Database ^2^ |
| Labour Force | World Bank Database ^2^ |
| Value of a Statistical Life (VSL) | Viscusi and Masterman (2017) ^8^ |
| Inflation Rate (2010-2020) | International Monetary Fund ^9^ |
| Population Estimates 2020-2050 | UN World Population Prospects (2022)^10^ |
| Schooling lost due to RHD | Koech and Ngeno (2014) ^11^ |
| Treatment coverage rates for surgical interventions | Zühlke et. al (2015) ^12^ |
| Treatment coverage rates for RHD and its complications | WHO Global Health Observatory – UHC Service Coverage Sub-index ^13^ |

**References**

1. Avenir Health. OneHealth Tool Intervention Assumptions. 2017. <https://avenirhealth.org/download/spectrum/manuals/treatment%20assumptions%202016%201%2010.pdf>.

2. World Bank. World Bank Open Data, 2024.

3. Network GBoDC. Global Burden of Disease Study 2019 (GBD 2019). Seattle, United States: Institute for Health Metrics and Evaluation (IHME), 2020, 2020.

4. Figueiredo ET, Azevedo L, Rezende ML, et al. Rheumatic Fever: A Disease without Color. *Arq Bras Cardiol* 2019;113(3):345-54. doi: 10.5935/abc.20190141 [published Online First: 20190729]

5. Economic Analysis and Evaluation Team, Department of Health Systems Governance and Financing WHO. WHO-CHOICE estimates of cost for inpatient and outpatient health service delivery (2010), 2021.

6. World Health Organization. Global Health Observatory - Life Expectancy: World Health Organization, 2024.

7. Kotseva K, Gerlier L, Sidelnikov E, et al. Patient and caregiver productivity loss and indirect costs associated with cardiovascular events in Europe. *European Journal of Preventive Cardiology* 2020;26(11):1150-57. doi: 10.1177/2047487319834770

8. Viscusi WK, Masterman CJ. Income Elasticities and Global Values of a Statistical Life. *Journal of Benefit-Cost Analysis* 2017;8(2):226-50. doi: 10.1017/bca.2017.12 [published Online First: 2017/07/27]

9. International Monetary Fund. Inflation rate, average consumer prices 2023 [Available from: <https://www.imf.org/external/datamapper/PCPIPCH@WEO/OEMDC/ADVEC/WEOWORLD/NGA> accessed 06/06/2023 2023.

10. UN World Population Prospects 2022: Population Division, Department of Economic and Social Affairs, United Nations; 2022 [Available from: <https://population.un.org/wpp/>.

11. Koech MM NT. Economic impact on families with children with rheumatic heart disease at a referral hospital, Kenya. *Basic Research Journal of Medicine and Clinical Sciences* 2014;3(8):74-79.

12. Zühlke L, Engel ME, Karthikeyan G, et al. Characteristics, complications, and gaps in evidence-based interventions in rheumatic heart disease: the Global Rheumatic Heart Disease Registry (the REMEDY study). *Eur Heart J* 2015;36(18):1115-22a. doi: 10.1093/eurheartj/ehu449 [published Online First: 20141125]

13. World Health Organization. Global Health Observatory - UHC service coverage index: World Health Organization, 2023.

**Supplementary Table 3. Number of patients with RHD, ARF, RHD comorbidities, and RHD related deaths at baseline (2020).**

| **Country** | **Number of patients (prevalent cases)** | | | | | | | **Number of deaths due to RHD** |
| --- | --- | --- | --- | --- | --- | --- | --- | --- |
|  | Acute rheumatic fever | Valve surgery | Heart failure | Atrial fibrillation | Infective endocarditis | Stroke | **Total number of patients with RHD** |  |
| **Bahrain** | 561 | 165 | 129 | 86 | 16 | 27 | **392** | 10 |
| **Kuwait** | 1,719 | 505 | 397 | 264 | 48 | 84 | **1,202** | 11 |
| **Oman** | 1,347 | 396 | 311 | 207 | 38 | 66 | **942** | 7 |
| **Qatar** | 2,714 | 797 | 626 | 418 | 76 | 133 | **1,898** | 7 |
| **Saudi Arabia** | 13,769 | 4,044 | 3,178 | 2,118 | 385 | 674 | **9,629** | 241 |
| **United Arab Emirates** | 77,047 | 22,629 | 17,780 | 11,853 | 2,155 | 3,772 | **53,879** | 134 |
| **Egypt** | 962,074 | 282,567 | 222,017 | 148,011 | 26,911 | 47,095 | **672,779** | 1,208 |
| **Iran, Islamic Republic of** | 653,014 | 191,794 | 150,695 | 100,464 | 18,266 | 31,966 | **456,653** | 1,050 |
| **Iraq** | 244,138 | 71,705 | 56,340 | 37,560 | 6,829 | 11,951 | **170,726** | 316 |
| **Jordan** | 2,590 | 761 | 598 | 398 | 72 | 127 | **1,811** | 27 |
| **Lebanon** | 2,678 | 787 | 618 | 412 | 75 | 131 | **1,873** | 48 |
| **Libya** | 57,449 | 16,873 | 13,257 | 8,838 | 1,607 | 2,812 | **40,174** | 55 |
| **Morocco** | 309,612 | 90,935 | 71,449 | 47,633 | 8,660 | 15,156 | **216,512** | 675 |
| **Occupied Palestinian territory** | 35,729 | 10,494 | 8,245 | 5,497 | 999 | 1,749 | **24,985** | 16 |
| **Syrian Arab Republic** | 119,948 | 35,230 | 27,680 | 18,454 | 3,355 | 5,872 | **83,880** | 236 |
| **Tunisia** | 5,187 | 1,523 | 1,197 | 798 | 145 | 254 | **3,627** | 125 |
| **Afghanistan** | 263,033 | 77,254 | 60,700 | 40,467 | 7,358 | 12,876 | **183,939** | 1,081 |
| **Djibouti** | 19,680 | 5,780 | 4,541 | 3,028 | 550 | 963 | **13,762** | 11 |
| **Pakistan** | 3,255,268 | 956,093 | 751,216 | 500,810 | 91,056 | 159,349 | **2,276,411** | 23,216 |
| **Somalia** | 223,227 | 65,563 | 51,514 | 34,343 | 6,244 | 10,927 | **156,103** | 389 |
| **Sudan** | 328,937 | 96,611 | 75,909 | 50,606 | 9,201 | 16,102 | **230,026** | 663 |
| **Yemen** | 287,451 | 84,426 | 66,335 | 44,223 | 8,041 | 14,071 | **201,015** | 621 |
| **Total** | **6,867,172** | **2,016,932** | **1,584,732** | **1,056,488** | **192,089** | **336,155** | **4,802,218** | **30,147** |

Source: Institute of Health and Metrics Global Burden of Health 2019, Figueiredo et al. (2019), UN World Population Prospects 2022.

**Supplementary Table 4. Average Treatment Cost (US$) data used for estimating the economic burden of RHD in EMRO**

| **Country** | **Average Treatment Cost Per Patient ($US)** | | | | | |
| --- | --- | --- | --- | --- | --- | --- |
|  | Acute rheumatic fever | Valve surgery | Heart failure | Atrial fibrillation | Infective endocarditis | Stroke |
| **Bahrain** | 168.5 | 2,999.8 | 1,303.2 | 409 | 1,642 | 3,052 |
| **Kuwait** | 327.8 | 5,944.8 | 2,755.5 | 864.7 | 3,471.8 | 6,453.1 |
| **Oman** | 129.6 | 2,285.3 | 947.7 | 297.4 | 1,194.1 | 2,219.5 |
| **Qatar** | 420.3 | 8,583.2 | 4,136.1 | 1,298 | 5,211.3 | 9,686.5 |
| **Saudi Arabia** | 123.8 | 2,135.2 | 857.4 | 269.1 | 1,080.3 | 2,007.9 |
| **United Arab Emirates** | 194.9 | 5,015.2 | 2,340.3 | 734.4 | 2,948.7 | 5,480.9 |
| **Egypt** | 46.8 | 1,723 | 270.4 | 84.8 | 340.6 | 633.1 |
| **Iran, Islamic Republic of** | 50.5 | 1,246.2 | 308 | 96.7 | 388.1 | 721.4 |
| **Iraq** | 28.8 | 689.7 | 135.5 | 42.5 | 170.7 | 317.3 |
| **Jordan** | 30.1 | 767.0 | 157.7 | 49.5 | 198.7 | 369.3 |
| **Lebanon** | 103.6 | 2,092.7 | 621.3 | 195.0 | 782.9 | 1,455.1 |
| **Libya** | 217 | 3,637.6 | 1,377.6 | 432.3 | 1,735.8 | 3,226.3 |
| **Morocco** | 21.1 | 594.4 | 108.8 | 34.1 | 137.0 | 254.7 |
| **Occupied Palestinian territory** | 0.6 | 434.8 | 21.9 | 6.9 | 27.6 | 51.3 |
| **Syrian Arab Republic** | 25.6 | 812.9 | 134.1 | 42.1 | 168.9 | 314.0 |
| **Tunisia** | 42.4 | 1,411.9 | 420.2 | 131.9 | 529.5 | 984.1 |
| **Afghanistan** | 9.5 | 656.8 | 54.9 | 17.2 | 69.2 | 128.5 |
| **Djibouti** | 11 | 541.7 | 60.7 | 19.1 | 76.5 | 142.2 |
| **Pakistan** | 14.2 | 932.7 | 89.9 | 28.2 | 113.3 | 210.6 |
| **Somalia** | 0.7 | 506.1 | 25.5 | 8 | 32.1 | 59.7 |
| **Sudan** | 40.4 | 1,165.1 | 151.1 | 47.4 | 190.4 | 353.9 |
| **Yemen** | 58.4 | 2,761.4 | 324.2 | 101.7 | 408.5 | 759.3 |
| **Total** | **114.2** | **2,413.6** | **938.3** | **294.4** | **1,182.2** | **2,197.4** |

Source: Institute of Health and Metrics Global Burden of Health 2019, Figueiredo et al. (2019), WHO-CHOICE, International Monetary Fund.

**Supplementary Table 5. Coverage rate (%) data used for estimating the economic burden of RHD in EMRO**

| **Country** | **Coverage Rate (%)** | | | | | |
| --- | --- | --- | --- | --- | --- | --- |
|  | Acute rheumatic fever | Valve surgery | Heart failure | Atrial fibrillation | Infective endocarditis | Stroke |
| **Bahrain** | 82 | 61 | 57 | 57 | 57 | 57 |
| **Kuwait** | 80 | 61 | 60 | 60 | 60 | 60 |
| **Oman** | 83 | 61 | 58 | 58 | 58 | 58 |
| **Qatar** | 80 | 61 | 62 | 62 | 62 | 62 |
| **Saudi Arabia** | 87 | 61 | 49 | 49 | 49 | 49 |
| **United Arab Emirates** | 89 | 61 | 61 | 61 | 61 | 61 |
| **Egypt** | 62 | 28 | 62 | 62 | 62 | 62 |
| **Iran, Islamic Republic of** | 55 | 61 | 69 | 69 | 69 | 69 |
| **Iraq** | 54 | 28 | 64 | 64 | 64 | 64 |
| **Jordan** | 62 | 61 | 58 | 58 | 58 | 58 |
| **Lebanon** | 83 | 61 | 54 | 54 | 54 | 54 |
| **Libya** | 57 | 61 | 52 | 52 | 52 | 52 |
| **Morocco** | 84 | 28 | 57 | 57 | 57 | 57 |
| **Occupied Palestinian territory e** | 54 | 28 | 60 | 60 | 60 | 60 |
| **Syrian Arab Republic** | 68 | 28 | 65 | 65 | 65 | 65 |
| **Tunisia** | 55 | 61 | 57 | 57 | 57 | 57 |
| **Afghanistan** | 33 | 11 | 65 | 65 | 65 | 65 |
| **Djibouti** | 35 | 28 | 58 | 58 | 58 | 58 |
| **Pakistan** | 38 | 28 | 55 | 55 | 55 | 55 |
| **Somalia** | 35 | 11 | 61 | 61 | 61 | 61 |
| **Sudan** | 43 | 28 | 54 | 54 | 54 | 54 |
| **Yemen** | 46 | 28 | 62 | 62 | 62 | 62 |

Source: WHO UHC Index, Zühlke et al. (2015)

**Supplementary Table 6. Reduction in future lifetime earnings for one additional year of school lost (%) per country**

| Country | Group | Reduction in future lifetime earnings for one additional year of school lost (%) |
| --- | --- | --- |
| Afghanistan | Low-income | 7.3% |
| Bahrain | High-income | 5.0% |
| Djibouti | Low-income | 8.3% |
| Egypt | Middle-income | 5.2% |
| Iran, Islamic Republic of | Middle-income | 7.8% |
| Iraq | Middle-income | 0.7% |
| Jordan | Middle-income | 5.0% |
| Kuwait | High-income | 5.0% |
| Lebanon | Middle-income | 5.0% |
| Libya | Middle-income | 6.9% |
| Morocco | Middle-income | 7.2% |
| Oman | High-income | 5.0% |
| Pakistan | Low-income | 7.7% |
| Occupied Palestinian territory | Middle-income | 2.7% |
| Qatar | High-income | 5.0% |
| Saudi Arabia | High-income | 5.0% |
| Somalia | Low-income | 8.3% |
| Sudan | Low-income | 7.7% |
| Syrian Arab Republic | Middle-income | 5.0% |
| Tunisia | Middle-income | 5.4% |
| United Arab Emirates | High-income | 5.0% |
| Yemen | Low-income | 5.0% |

Source: OneHealth Tool

**Supplementary Table 7. Cumulated number of RHD-related deaths in 2020, by 2030, 2040, and 2050 (in thousands) with uncertainty intervals**

| **Year** | **High-income** | **Middle-income** | **Low-income** | **Total** |
| --- | --- | --- | --- | --- |
| 2020 | 0.4  (0.2- 0.7) | 3.8  (2.5-5.9) | 26.0  (16.9-37.3) | **30.1**  **(20.0-44.0)** |
| 2020-2030 | 5.5  (3.1-9.1) | 48.7  (32.6-75.8) | 330.1  (214.2-475.0) | **384.4**  **(249.9-559.9)** |
| 2020-2040 | 13.5  (7.7-21.8) | 111.7  (74.9-171.7) | 733.8  (476.4-1,056.9) | **859.0**  **(559.0-1,250.0)** |
| 2020-2050 | 25.4  (14.6-40.7) | 197.2  (132.3-299.5) | 1,259.2  (817.9-1,815.6) | **1,481.7**  **(964.8-2,155.7)** |

**Supplementary Table 8. Cumulated number of prevalent cases of RHD in 2020, by 2030, 2040, and 2050 (in thousands) with uncertainty intervals**

| **Year** | **High-income** | **Middle-income** | **Low-income** | **Total** |
| --- | --- | --- | --- | --- |
| 2020 | 68  (53-98) | 1,673  (1,198-2,291) | 3,061  (1,737-3,435) | **4,802**  **(2,988-5,825)** |
| 2020-2030 | 811  (624-1,142) | 19,993 (14,394-27,440) | 37,343  (21,854-43,006) | **58,147**  **(36,873-71,588)** |
| 2020-2040 | 1,667  (1,264-2,314) | 40,924 (29,492-55,983) | 78,372 (47,309-92,522) | **120,963**  **(78,065-150,819)** |
| 2020-2050 | 2,633 (1,975-3,615) | 63,849 (46,049-87,101) | 125,754  (77,998-151,672) | **192,235**  **(126,022-242,389)** |

**Supplementary Table 9. Estimated economic burden (US$) of RHD per country in 2020 in EMRO (in millions, US$) with uncertainty intervals**

|  | **Direct Healthcare Costs** | **Foregone Productivity due to Absenteeism** | **Future Lifetime Earnings Lost** | **Cost of Premature Deaths** | **Total Economic Burden (2020)** | **Total Economic Burden (% of GDP)** |
| --- | --- | --- | --- | --- | --- | --- |
| **Bahrain** | 0.56 (0.38 - 0.78) | 1.27 (0.85 - 1.82) | 0.11 (0.04 - 0.2) | 9.33 (0.5 - 12.84) | 11.27 (1.77 - 15.65) | 0.03 (0.01-0.05) |
| **Kuwait** | 3.51 (2.36 - 4.94) | 1.25 (0.81 - 1.81) | 0.15 (0.06 - 0.31) | 30.23 (21.93 - 54.31) | 35.14 (25.16 - 61.36) | 0.03 (0.02-0.06) |
| **Oman** | 1.02 (0.67 - 1.46) | 0.95 (0.61 - 1.39) | 0.13 (0.05 - 0.26) | 4.29 (3.27 - 12.91) | 6.39 (4.6 - 16.02) | 0.01 (0.01-0.02) |
| **Qatar** | 8.1 (1.69 - 3.64) | 7.59 (1.58 - 3.49) | 0.29 (0.12 - 0.58) | 39.75 (0 - 47.75) | 55.73 (3.39 - 55.46) | 0.04 (0-0.04) |
| **Saudi Arabia** | 9.25 (6.47 - 12.72) | 1.22 (0.83 - 1.71) | 0.17 (0.05 - 0.31) | 448.87 (256.25 - 711.13) | 459.51 (263.6 - 725.88) | 0.06 (0.04-0.1) |
| **United Arab Emirates** | 130.2 (107.74 - 194.08) | 41.48 (36.93 - 72.75) | 10.69 (5.53 - 17.75) | 357.16 (179.24 - 676.34) | 539.53 (329.44 - 960.92) | 0.15 (0.09-0.27) |
| **Egypt** | 234.02 (149.32 - 289.02) | 8.75 (5.23 - 10.45) | 3.06 (1.55 - 5.05) | 263.5 (164.7 - 575.51) | 509.33 (320.8 - 880.03) | 0.13 (0.08-0.23) |
| **Iran, Islamic Republic of** | 224.01 (171.64 - 313.85) | 3.65 (2.93 - 5.73) | 3.09 (1.51 - 4.75) | 391.02 (325.3 - 462.59) | 621.77 (501.37 - 786.91) | 0.26 (0.21-0.33) |
| **Iraq** | 26.81 (23.58 - 46.93) | 7.06 (6.31 - 12.64) | 0.51 (0.26 - 0.84) | 122.51 (78.48 - 184.68) | 156.88 (108.63 - 245.09) | 0.09 (0.06-0.14) |
| **Jordan** | 0.51 (0.34 - 0.73) | 0.19 (0.13 - 0.28) | 0.03 (0.01 - 0.06) | 7.67 (4.38 - 10.13) | 8.4 (4.86 - 11.21) | 0.02 (0.01-0.03) |
| **Lebanon** | 1.62 (1.11 - 2.24) | 0.39 (0.26 - 0.56) | 0.06 (0.02 - 0.11) | 17.91 (6.35 - 33.59) | 19.99 (7.74 - 36.5) | 0.06 (0.02-0.12) |

**Supplementary Table 10. Cumulated economic burden (in millions, US$) in 2020, by 2030, 2040, and 2050 with uncertainty intervals**

| **Year** | **High-income** | **Middle-income** | **Low-income** | **Total** |
| --- | --- | --- | --- | --- |
| 2020 | 1,107  (628 – 1,835) | 1,660  (1155.9 - 2464.72) | 3,031  (1,894 – 4,217) | **5,798**  **(3,678 – 8,517)** |
| 2020-2030 | 12,236  (7,035 – 20,135) | 17,579  (12,276 – 26,021) | 32,611  (20,481 – 45,560) | **62,426**  **(39,793 – 91,715)** |
| 2020-2040 | 23,465  (13,586 – 38,417) | 32,181  (22,494 – 47,451) | 60,635  (38,239 – 85,059) | **116,280**  **(74,319 – 170,927)** |
| 2020-2050 | 34,312  (19,818 – 55,962) | 45,184  (31,601 – 66,411) | 86,476  (54,726 – 121,736) | **165,972**  **(106,146 – 244,109)** |

**Supplementary Table 11. Estimated accumulated economic burden (in millions, US$) from 2020–2050 of RHD by country in the EMR with uncertainty intervals.**

|  | **Cost of Premature Deaths** | **Foregone Productivity due to Absenteeism** | **Direct Healthcare Costs** | **Future Lifetime Earnings Lost** | **Total** |
| --- | --- | --- | --- | --- | --- |
| **Bahrain** | 237.28 (121.41 - 335.64) | 39.71 (26.69 - 56.53) | 17.81 (12.26 - 24.62) | 2.46 (0.75 - 4.92) | 297.25 (161.1 - 421.7) |
| **Kuwait** | 1,141.12 (770.6 – 1,735.46) | 38.73 (25.46 - 55.66) | 120.71 (82.98 - 166.2) | 3.73 (1.26 - 7.41) | 1,304.29 (880.3 – 1,964.74) |
| **Oman** | 208.94 (135.17 - 396.26) | 29.53 (19.22 - 42.72) | 31.44 (20.86 - 44.39) | 3.07 (0.98 - 6.27) | 272.99 (176.23 - 489.64) |
| **Qatar** | 966.34 (360.55 – 1,440.57) | 244.09 (49.96 - 109.5) | 222.6 (53.16 - 111.82) | 6.86 (2.38 - 13.85) | 1,439.89 (466.05 – 1,675.74) |
| **Saudi Arabia** | 13,360.04 (7,948.12 – 20,510.91) | 38.18 (26.35 - 53.19) | 323.17 (228.93 - 437.82) | 3.91 (1.46 - 7.55) | 13,725.29 (8,204.86 – 21,009.46) |
| **United Arab Emirates** | 12,828.56 (6,385.68 – 23,706.54) | 1,224.58 (1,061.67 – 2,049.47) | 2,959.46 (2,348.21 – 4,213.83) | 259.54 (134.18 - 430.78) | 17,272.14 (9,929.74 – 30,400.62) |
| **Egypt** | 7,504.12 (4,618.52 – 15,825.87) | 240.25 (150.58 - 294.6) | 5,807.85 (3,808.01 – 7,309.35) | 74.47 (37.83 - 122.88) | 13,626.69 (8,614.94 – 2,3552.7) |
| **Iran, Islamic Republic of** | 11,585.74 (9,634.73 – 13,651.27) | 106.48 (84.37 - 161.47) | 4,944.22 (3,721.68 – 6,737.78) | 75.32 (36.52 - 115.19) | 16,711.75 (13,477.3 – 20,665.72) |
| **Iraq** | 4,033.67 (2,570 – 5,966.58) | 209.14 (181.05 - 355.58) | 784.73 (668.03 – 1,307.75) | 12.43 (6.41 - 20.49) | 5,039.97 (3,425.49 – 7,650.4) |
| **Jordan** | 235.32 (145.65 - 323.97) | 5.92 (3.89 - 8.57) | 14.71 (9.86 - 20.79) | 0.79 (0.26 - 1.55) | 256.73 (159.66 - 354.88) |
| **Lebanon** | 415.99 (152.7 - 712.97) | 12.14 (8.22 - 17.14) | 36.9 (25.17 - 50.7) | 1.38 (0.5 - 2.65) | 466.41 (186.59 - 783.47) |
| **Libya** | 696.67 (371.37 – 1,125.07) | 430.29 (268.21 - 525.79) | 1416.03 (962.29 - 1824.31) | 173.31 (88.87 - 284.98) | 2,716.31 (1,690.73 – 3,760.15) |
| **Morocco** | 3,449.1 (2,114.93 – 5,353.63) | 197.87 (124.07 - 244.45) | 643.07 (440.67 - 837.16) | 88.8 (45.23 - 146.9) | 4,378.84 (2,724.91 – 6,582.13) |
| **Occupied Palestinian territory** | 88.13 (55.48 - 120.54) | 203.45 (144.41 - 280.66) | 42.68 (30.32 - 58.86) | 36.3 (18.68 - 60.26) | 370.56 (248.88 - 520.32) |
| **Syrian Arab Republic** | 332.5 (192.78 - 516.68) | 24.63 (21.62 - 42.18) | 503.83 (422.68 - 807.62) | 10.16 (5.2 - 16.82) | 871.12 (642.27 – 1,383.31) |
| **Tunisia** | 684.18 (388.27 – 1,073.58) | 6.29 (4.23 - 8.88) | 54.27 (37.65 - 74.03) | 0.73 (0.22 - 1.47) | 745.46 (430.38 – 1,157.96) |
| **Afghanistan** | 1,445.56 (733.79 – 2,527.3) | 36.25 (24.95 - 50.97) | 316.39 (215.61 - 448.24) | 19.92 (10.13 - 33.48) | 1,818.11 (984.48 – 3,059.99) |
| **Djibouti** | 44.49 (15.97 - 96.63) | 305.01 (215.5 - 419.22) | 30.16 (21.55 - 41.12) | 267.66 (138.02 - 432.49) | 647.31 (391.03 - 989.46) |
| **Pakistan** | 66,063.93 (42,937.3 – 93,529.46) | 141.12 (79.42 - 151.94) | 8,450.64 (4,868.16 – 9,287.45) | 62.71 (34.71 - 99.48) | 74,718.4 (47,919.58 – 103,068.32) |
| **Somalia** | 336.93 (151.77 - 654.01) | 49.12 (37.78 - 76.81) | 169.38 (134.99 - 282.34) | 53.57 (27.44 - 88.24) | 609 (351.98 – 1,101.4) |
| **Sudan** | 2,764.11 (1,352.5 – 4,926.01) | 42.02 (29.15 - 58.18) | 1,429.16 (944.45 – 1,857.76) | 70.56 (11.96 - 38.91) | 4,305.86 (2,338.06 – 6,880.85) |
| **Yemen** | 1,570.4 (801.07 – 2,733.53) | 38.86 (26.9 - 53.94) | 2,754.3 (1,906.19 – 3,825.24) | 14.18 (7.19 - 23.63) | 4,377.74 (2,741.36 – 6,636.35) |
| **Total** | **129,993.11 (81,958.35 – 197,262.48)** | **3,663.66 (2,613.69 – 5,117.43)** | **31,073.49 (20,963.7 – 39,769.22)** | **1,241.85 (610.17 – 1,960.19)** | **165,972.1 (106,145.9 – 244,109.32)** |

**Supplementary Table 12. Estimated direct costs (in millions, US$) of RHD in 2020 per country in EMRO by intervention type with uncertainty intervals.**

| **Country** | **Direct cost** | | | | | |  |
| --- | --- | --- | --- | --- | --- | --- | --- |
|  | **Acute rheumatic fever** | **Stroke** | **Valve surgery** | **Heart failure** | **Infective endocarditis** | **Atrial fibrillation** | **Total** |
| **Bahrain** | 0.08 (0.05 - 0.11) | 0.05 (0.03 - 0.07) | 0.3 (0.21 - 0.42) | 0.1 (0.07 - 0.14) | 0.01 (0.01 - 0.02) | 0.02 (0.01 - 0.03) | 0.56 (0.38 - 0.78) |
| **Kuwait** | 0.45 (0.3 - 0.63) | 0.33 (0.22 - 0.46) | 1.83 (1.23 - 2.58) | 0.66 (0.45 - 0.93) | 0.1 (0.07 - 0.14) | 0.14 (0.09 - 0.19) | 3.51 (2.36 - 4.94) |
| **Oman** | 0.14 (0.09 - 0.21) | 0.09 (0.06 - 0.12) | 0.55 (0.36 - 0.79) | 0.17 (0.11 - 0.25) | 0.03 (0.02 - 0.04) | 0.04 (0.02 - 0.05) | 1.02 (0.67 - 1.46) |
| **Qatar** | 0.91 (0.19 - 0.41) | 0.81 (0.17 - 0.36) | 4.17 (0.87 - 1.87) | 1.63 (0.34 - 0.73) | 0.25 (0.05 - 0.11) | 0.33 (0.07 - 0.15) | 8.1 (1.69 - 3.64) |
| **Saudi Arabia** | 1.48 (1.04 - 2.04) | 0.67 (0.47 - 0.92) | 5.27 (3.68 - 7.24) | 1.35 (0.94 - 1.86) | 0.2 (0.14 - 0.28) | 0.28 (0.19 - 0.38) | 9.25 (6.47 - 12.72) |
| **United Arab Emirates** | 13.35 (11.05 - 19.9) | 12.79 (10.58 - 19.06) | 69.23 (57.29 - 103.19) | 25.69 (21.26 - 38.3) | 3.88 (3.21 - 5.78) | 5.26 (4.35 - 7.84) | 130.2 (107.74 - 194.08) |
| **Egypt** | 27.89 (17.79 - 34.44) | 18.75 (11.96 - 23.16) | 136.32 (86.98 - 168.36) | 37.67 (24.03 - 46.52) | 5.68 (3.63 - 7.02) | 7.71 (4.92 - 9.53) | 234.02 (149.32 - 289.02) |
| **Iran, Islamic Republic of** | 18.12 (13.88 - 25.39) | 16.14 (12.37 - 22.61) | 145.8 (111.71 - 204.27) | 32.42 (24.84 - 45.42) | 4.89 (3.75 - 6.85) | 6.64 (5.09 - 9.3) | 224.01 (171.64 - 313.85) |
| **Iraq** | 3.79 (3.34 - 6.64) | 2.46 (2.17 - 4.31) | 13.85 (12.18 - 24.24) | 4.95 (4.35 - 8.66) | 0.75 (0.66 - 1.31) | 1.01 (0.89 - 1.77) | 26.81 (23.58 - 46.93) |
| **Jordan** | 0.05 (0.03 - 0.07) | 0.03 (0.02 - 0.04) | 0.36 (0.24 - 0.51) | 0.06 (0.04 - 0.08) | 0.01 (0.01 - 0.01) | 0.01 (0.01 - 0.02) | 0.51 (0.34 - 0.73) |
| **Lebanon** | 0.23 (0.16 - 0.32) | 0.1 (0.07 - 0.14) | 1 (0.68 - 1.39) | 0.21 (0.14 - 0.29) | 0.03 (0.02 - 0.04) | 0.04 (0.03 - 0.06) | 1.62 (1.11 - 2.24) |
| **Libya** | 7.1 (4.65 - 8.92) | 4.79 (3.13 - 6.02) | 37.44 (24.52 - 47.07) | 9.61 (6.3 - 12.08) | 1.45 (0.95 - 1.82) | 1.97 (1.29 - 2.48) | 62.36 (40.84 - 78.39) |
| **Morocco** | 5.48 (3.65 - 7.01) | 2.23 (1.49 - 2.85) | 15.14 (10.09 - 19.35) | 4.48 (2.99 - 5.73) | 0.68 (0.45 - 0.86) | 0.92 (0.61 - 1.17) | 28.93 (19.29 - 36.98) |
| **Occupied Palestinian territory** | 0.01 (0.01 - 0.02) | 0.05 (0.04 - 0.08) | 1.28 (0.9 - 1.78) | 0.11 (0.08 - 0.15) | 0.02 (0.01 - 0.02) | 0.02 (0.02 - 0.03) | 1.49 (1.05 - 2.08) |
| **Syrian Arab Republic** | 2.09 (1.88 - 3.72) | 1.22 (1.1 - 2.17) | 8.02 (7.23 - 14.31) | 2.44 (2.2 - 4.36) | 0.37 (0.33 - 0.66) | 0.5 (0.45 - 0.89) | 14.63 (13.19 - 26.1) |
| **Tunisia** | 0.12 (0.08 - 0.17) | 0.14 (0.1 - 0.2) | 1.31 (0.9 - 1.8) | 0.29 (0.2 - 0.4) | 0.04 (0.03 - 0.06) | 0.06 (0.04 - 0.08) | 1.97 (1.36 - 2.71) |
| **Afghanistan** | 0.82 (0.55 - 1.18) | 1.09 (0.73 - 1.56) | 5.63 (3.79 - 8.06) | 2.19 (1.47 - 3.14) | 0.33 (0.22 - 0.47) | 0.45 (0.3 - 0.64) | 10.52 (7.08 - 15.05) |
| **Djibouti** | 0.08 (0.05 - 0.1) | 0.08 (0.06 - 0.11) | 0.88 (0.62 - 1.2) | 0.16 (0.11 - 0.22) | 0.02 (0.02 - 0.03) | 0.03 (0.02 - 0.05) | 1.25 (0.89 - 1.72) |
| **Pakistan** | 17.55 (9.17 - 17.77) | 18.72 (9.78 - 18.96) | 249.69 (130.46 - 252.9) | 37.61 (19.65 - 38.09) | 5.67 (2.97 - 5.75) | 7.7 (4.02 - 7.8) | 336.94 (176.05 - 341.28) |
| **Somalia** | 0.05 (0.04 - 0.09) | 0.4 (0.33 - 0.69) | 3.65 (2.94 - 6.21) | 0.81 (0.65 - 1.38) | 0.12 (0.1 - 0.21) | 0.17 (0.13 - 0.28) | 5.21 (4.2 - 8.86) |
| **Sudan** | 5.71 (3.74 - 7.45) | 3.12 (2.04 - 4.07) | 31.52 (20.63 - 41.11) | 6.27 (4.1 - 8.18) | 0.95 (0.62 - 1.23) | 1.28 (0.84 - 1.67) | 48.85 (31.98 - 63.71) |
| **Yemen** | 7.71 (5.26 - 10.82) | 6.72 (4.58 - 9.42) | 65.28 (44.54 - 91.56) | 13.5 (9.21 - 18.93) | 2.04 (1.39 - 2.86) | 2.76 (1.89 - 3.88) | 98.01 (66.87 - 137.47) |
| **Total** | 113.22 (77.03 - 147.4) | 90.79 (61.5 - 117.4) | 798.51 (522.07 - 1000.22) | 182.37 (123.54 - 235.82) | 27.52 (18.64 - 35.58) | 37.35 (25.3 - 48.3) | 1249.77 (828.08 - 1584.74) |

**Supplementary Table 13. Estimated direct costs (US$) of RHD in EMRO in 2020 by type of costs**

|  | **Direct cost** | **Government Health Expenditure** | **Private Health Expenditure** | **Out of pocket Expenditure** |
| --- | --- | --- | --- | --- |
| **Bahrain** | 558,921 | 320,262 | 238,659 | 148,673 |
| **Kuwait** | 3,511,279 | 3,068,858 | 442,421 | 319,526 |
| **Oman** | 1,016,856 | 897,883 | 118,972 | 46,775 |
| **Qatar** | 8,098,656 | 6,049,696 | 2,048,960 | 769,372 |
| **Saudi Arabia** | 9,253,483 | 5,774,173 | 3,479,309 | 1,480,557 |
| **United Arab Emirates** | 130,198,798 | 72,390,532 | 57,808,266 | 14,582,265 |
| **Egypt** | 234,024,809 | 68,803,294 | 165,221,515 | 138,776,711 |
| **Iran, Islamic Republic of** | 224,009,866 | 102,820,529 | 121,189,338 | 82,883,651 |
| **Iraq** | 26,805,762 | 13,027,600 | 13,778,161 | 12,008,981 |
| **Jordan** | 506,718 | 272,107 | 234,610 | 153,029 |
| **Lebanon** | 1,623,332 | 827,899 | 795,433 | 590,893 |
| **Libya** | 62,356,466 | 39,471,643 | 22,884,823 | 27,561,558 |
| **Morocco** | 28,927,849 | 11,686,851 | 17,240,998 | 12,149,696 |
| **Occupied Palestinian territory** | 1,492,473 | 676,090 | 816,383 | 668,628 |
| **Syrian Arab Republic** | 14,629,626 | 6,773,517 | 7,856,109 | 7,899,998 |
| **Tunisia** | 1,970,661 | 1,139,042 | 831,619 | 717,321 |
| **Afghanistan** | 10,520,907 | 2,272,516 | 8,248,391 | 7,870,690 |
| **Djibouti** | 1,252,343 | 869,126 | 383,217 | 333,123 |
| **Pakistan** | 336,944,098 | 121,636,820 | 215,307,279 | 186,667,031 |
| **Somalia** | 5,208,061 | 937,451 | 4,270,610 | 4,218,529 |
| **Sudan** | 48,847,558 | 14,898,505 | 33,949,053 | 25,889,206 |
| **Yemen** | 98,007,219 | 17,641,299 | 80,365,919 | 79,385,847 |
| **Total** | **1,249,765,739** | **492,255,693** | **757,510,046** | **605,122,061** |

* Out of pocket Expenditure is part of the private Health Expenditure.

**Supplementary Table 14. Sensitivity analysis of total economic burden in 2020, and economic burden as percentage of 2020 GDP using various discount rates**

| **3% Discount Rate** | | |  |
| --- | --- | --- | --- |
| **Country Group** | **Economic Burden (2020)** | **% of 2020 GDP** | **Economic Burden (2020-2050)** |
| High-income | 1,107,579,189 | 0.08% | 34,311,847,860 |
| Middle-income | 1,659,851,357 | 0.15% | 45,183,839,073 |
| Low-income | 3,030,746,973 | 0.80% | 86,476,417,597 |
| **Total** | **5,798,177,519** | **0.20%** | **165,972,104,530** |
| **5% Discount Rate** | | | |
| **Country Group** | **Economic Burden (2020)** | **% of 2020 GDP** | **Economic Burden (2020-2050)** |
| High-income | 901,876,148 | 0.06% | 21,668,757,644 |
| Middle-income | 1,443,065,753 | 0.13% | 30,404,715,653 |
| Low-income | 2,466,330,884 | 0.65% | 54,207,256,773 |
| **Total** | **4,811,272,785** | **0.16%** | **106,280,730,070** |
| **7% Discount Rate** | | | |
| **Country Group** | **Economic Burden (2020)** | **% of 2020 GDP** | **Economic Burden (2020-2050)** |
| High-income | 765,396,539 | 0.05% | 14,573,724,729 |
| Middle-income | 1,300,042,543 | 0.12% | 21,812,327,554 |
| Low-income | 2,097,888,684 | 0.55% | 36,594,250,959 |
| **Total** | **4,163,327,765** | **0.14%** | **72,980,303,243** |

**Supplementary Table 15. Structure of the estimated economic burden of RHD in 2020 in EMRO**

| **Country** | **Direct cost** | | | **Indirect cost** | | | |
| --- | --- | --- | --- | --- | --- | --- | --- |
|  | **Direct cost %** | Government % | Out-of-pocket % | **Indirect cost %** | Absenteeism % | Future productivity lost % | Premature death % |
| **Bahrain** | **4.96%** | 2.8% | 1.3% | **95%** | 11% | 1.0% | 82.8% |
| **Kuwait** | **9.99%** | 8.7% | 0.9% | **90%** | 4% | 0.4% | 86.0% |
| **Oman** | **15.92%** | 14.1% | 0.7% | **84%** | 15% | 2.0% | 67.2% |
| **Qatar** | **14.53%** | 10.9% | 1.4% | **85%** | 14% | 0.5% | 71.3% |
| **Saudi Arabia** | **2.01%** | 1.3% | 0.3% | **98%** | 0% | 0.0% | 97.7% |
| **United Arab Emirate** | **24.13%** | 13.4% | 2.7% | **76%** | 8% | 2.0% | 66.2% |
| **Egypt** | **45.95%** | 13.5% | 27.2% | **54%** | 2% | 0.6% | 51.7% |
| **Iran, Islamic Republic of** | **36.03%** | 16.5% | 13.3% | **64%** | 1% | 0.5% | 62.9% |
| **Iraq** | **17.09%** | 8.3% | 7.7% | **83%** | 4% | 0.3% | 78.1% |
| **Jordan** | **6.03%** | 3.2% | 1.8% | **94%** | 2% | 0.4% | 91.3% |
| **Lebanon** | **8.12%** | 4.1% | 3.0% | **92%** | 2% | 0.3% | 89.6% |
| **Libya** | **55.67%** | 35.2% | 24.6% | **44%** | 14% | 6.4% | 24.0% |
| **Morocco** | **17.46%** | 7.1% | 7.3% | **83%** | 4% | 2.2% | 76.0% |
| **Occupied Palestinian territory** | **11.67%** | 5.3% | 5.2% | **88%** | 56% | 11.7% | 20.8% |
| **Syrian Arab Republic** | **56.09%** | 26.0% | 30.3% | **44%** | 3% | 1.6% | 39.1% |
| **Tunisia** | **7.32%** | 4.2% | 2.7% | **93%** | 1% | 0.1% | 91.8% |
| **Afghanistan** | **18.62%** | 4.0% | 13.9% | **81%** | 2% | 1.4% | 77.7% |
| **Djibouti** | **5.09%** | 3.5% | 1.4% | **95%** | 44% | 44.5% | 6.1% |
| **Pakistan** | **12.79%** | 4.6% | 7.1% | **87%** | 0% | 0.1% | 86.9% |
| **Somalia** | **26.10%** | 4.7% | 21.1% | **74%** | 9% | 11.0% | 54.4% |
| **Sudan** | **33.71%** | 10.3% | 17.9% | **66%** | 1% | 2.0% | 63.3% |
| **Yemen** | **65.49%** | 11.8% | 53.0% | **35%** | 1% | 0.4% | 33.2% |
| **Total** | **22.49%** | **9.71%** | **11.13%** | **78%** | **9%** | **4%** | **64%** |

**Supplementary Table 16. Estimated direct costs (US$) of RHD in EMR in 2020 by type of costs per country group**

| **Country Group** | **Direct cost** | **Government Health Expenditure** | **Private Health Expenditure** | **Out of pocket Expenditure** |
| --- | --- | --- | --- | --- |
| **High-income** | 152,637,993 | 88,501,404 | 64,136,587 | 17,347,168 |
| **Middle-income** | 596,347,562 | 245,498,572 | 350,848,989 | 283,410,466 |
| **Low-income** | 500,780,186 | 158,255,717 | 342,524,469 | 304,364,426 |
| **Total** | **1,249,765,739** | **492,255,693** | **757,510,046** | **605,122,061** |
